# Supplementary material for: Characterising proteolysis during SARS-CoV-2 infection identifies viral cleavage sites and cellular targets with therapeutic potential
Source: Nat Commun. 2021 Sep 21;12:5553. doi: 10.1038/s41467-021-25796-w (PMC8455558; doi:10.1038/s41467-021-25796-w)
Supplement: Supplementary file 1 — Supplementary Information [file 41467_2021_25796_MOESM1_ESM.pdf]

Supplementary Information

Characterising proteolysis during SARS-CoV-2 infection identifies viral cleavage sites and cellular targets with therapeutic potential

Bjoern Meyer[1] ,Jeanne Chiaravalli[2] ,Stacy Gellenoncourt[3], Philip Brownridge[4], Dominic P. Bryne[5], Leonard A. Daly[4], Arturas Grauslys[6], Marius Walter[7], Fabrice Agou[2], Lisa A. Chakrabarti[3], Charles S. Craik[8], Claire E. Eyers[4], Patrick A. Eyers[5], Yann Gambin[9], Andrew R. Jones[5], Emma Sierecki[9], Eric Verdin[7], Marco Vignuzzi[1] & Edward Emmott[4*]

*Corresponding author: Dr Edward Emmott: e.emmott@liverpool.ac.uk

Supplementary Figures 1-20

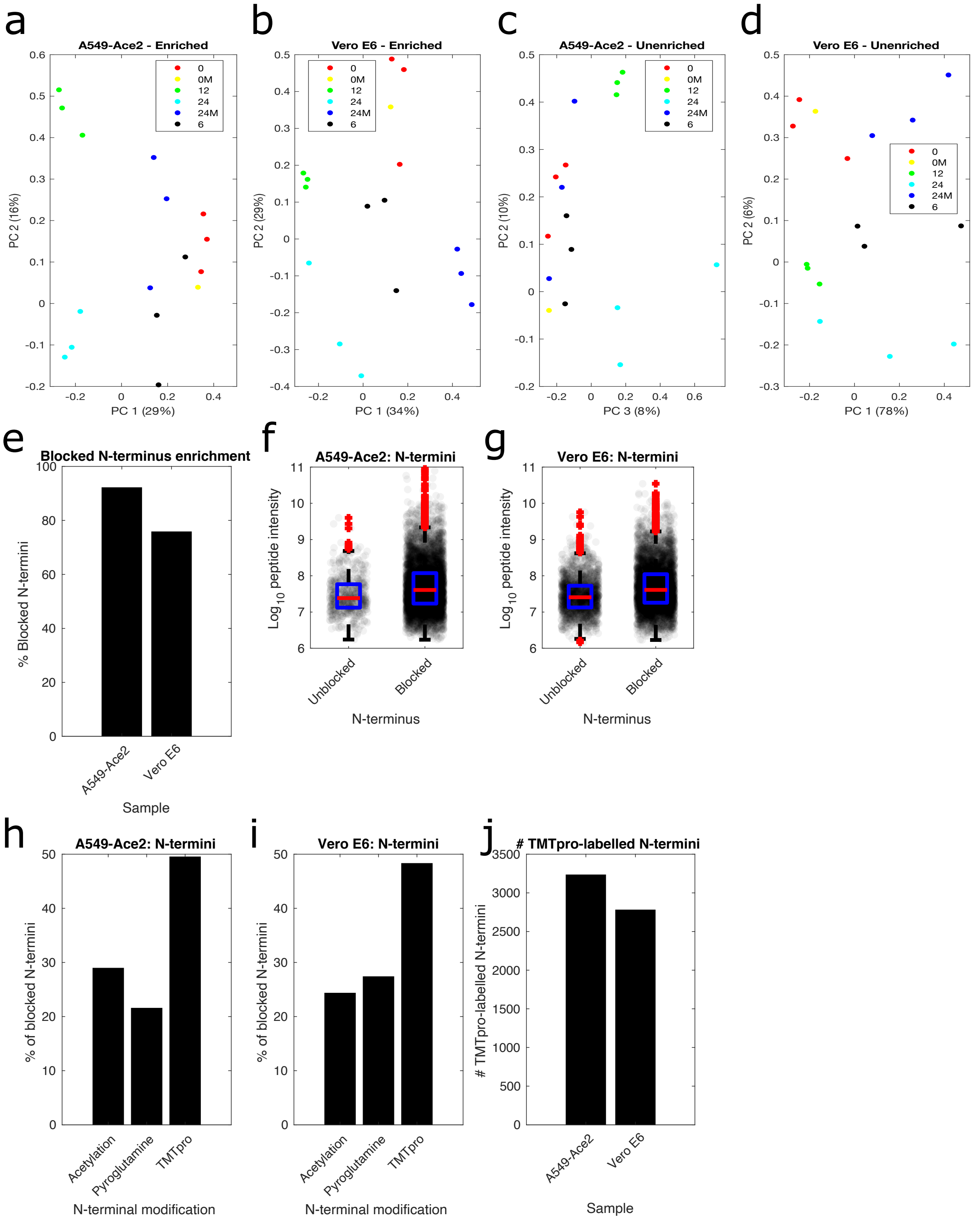

Supplementary Figure 1. Quality control of the proteomic datasets, both pre- and post-enrichment for N-termini. a-d) Principal component analysis separates infected from mock cells and shows reproducible clustering of biological replicates. e) Enrichment results in a majority of peptide identifications belonging to blocked N-termini, with blocked N-termini most abundant in both f) A549-Ace2 and g) Vero E6 cells. In both cell lines, TMTpro-labelled N-termini are the most abundant enriched N-termini h), i). j) over 2700 TMTpro-labelled N-termini were identified from each dataset.

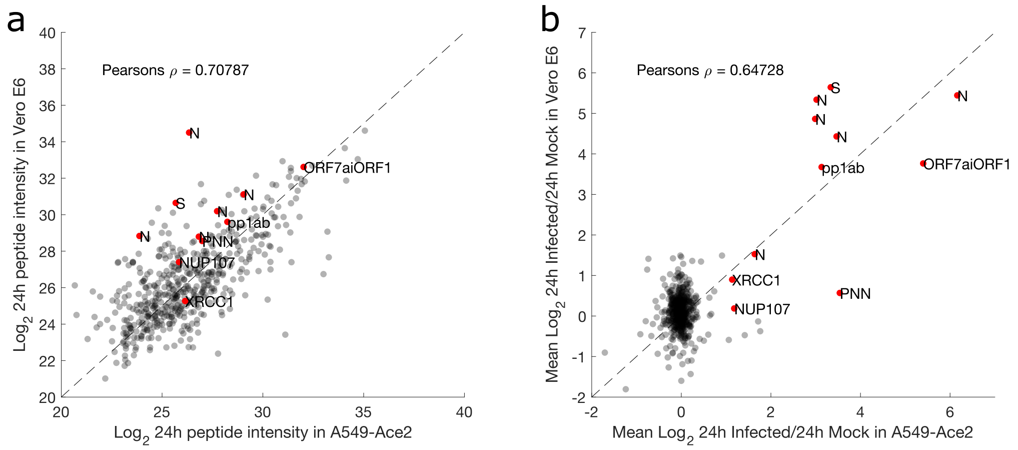

Supplementary Figure 2. Correlations of neo-N-termini shared between both the A549-Ace2 and Vero E6 datasets. a) Mean peptide intensity correlations for the 24h infected A549-Ace2 and Vero E6 samples. b) Log_2 24h infected/mock correlations for the A549-Ace2 and Vero E6 samples. Mean values (n=3) are shown, specific neo-N-termini mentioned by name in the text are highlighted in red and labelled. 497 neo-N-termini were quantifiable and common to both datasets.

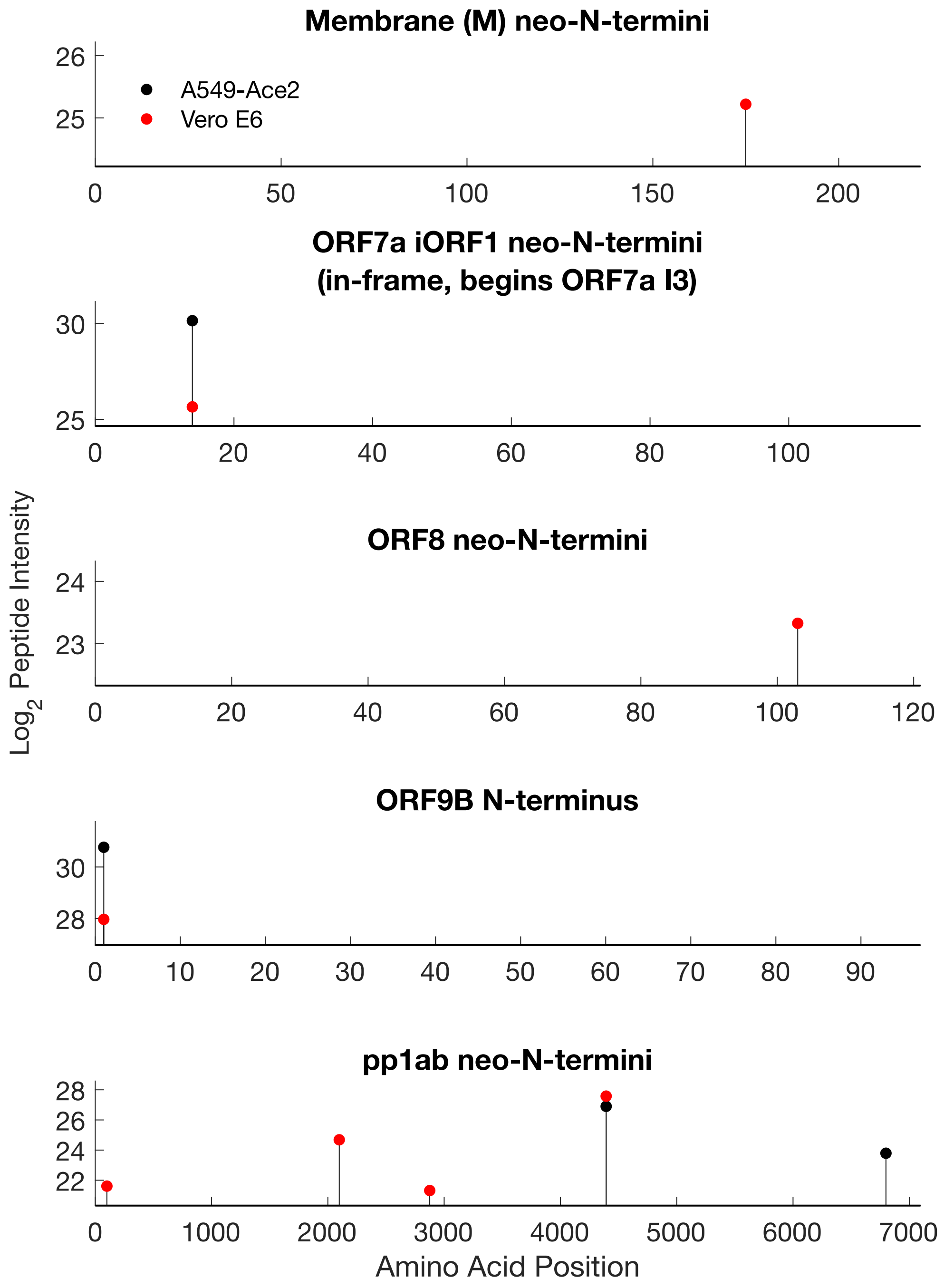

Supplementary Figure 3. Viral N-termini and neo-N-termini identified from the viral M, ORF7A/ORF7A iORF1, ORF8, ORF9B and pp1ab replicase. Please note that ORF7A iORF1 is an N-terminally truncated form of ORF7A that initiates at isoleucine 3 in the ORF7A sequence. The indicated neo-N-terminus beginning at amino acid 14, would therefore be amino acid 16 in ORF7A.

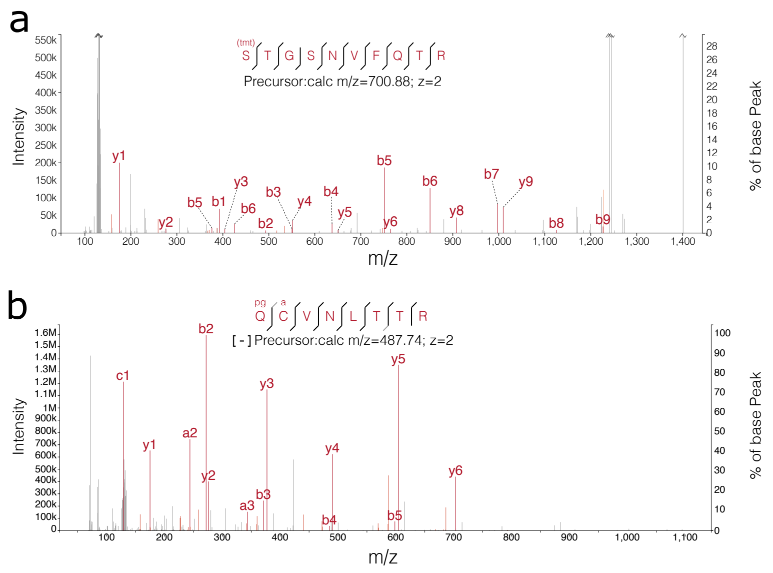

Supplementary Figure 4. Tandem Mass spectra for a) SARS-CoV-2 spike 637 neo-N-terminus and b) pyroglutamate-modified SARS-CoV-2 spike signal peptide cleavage site beginning Q14. b/y ions are shown in both. a/c ions are also included in b) due to the fragmentation properties of pyroglutamate-containing peptides (78).

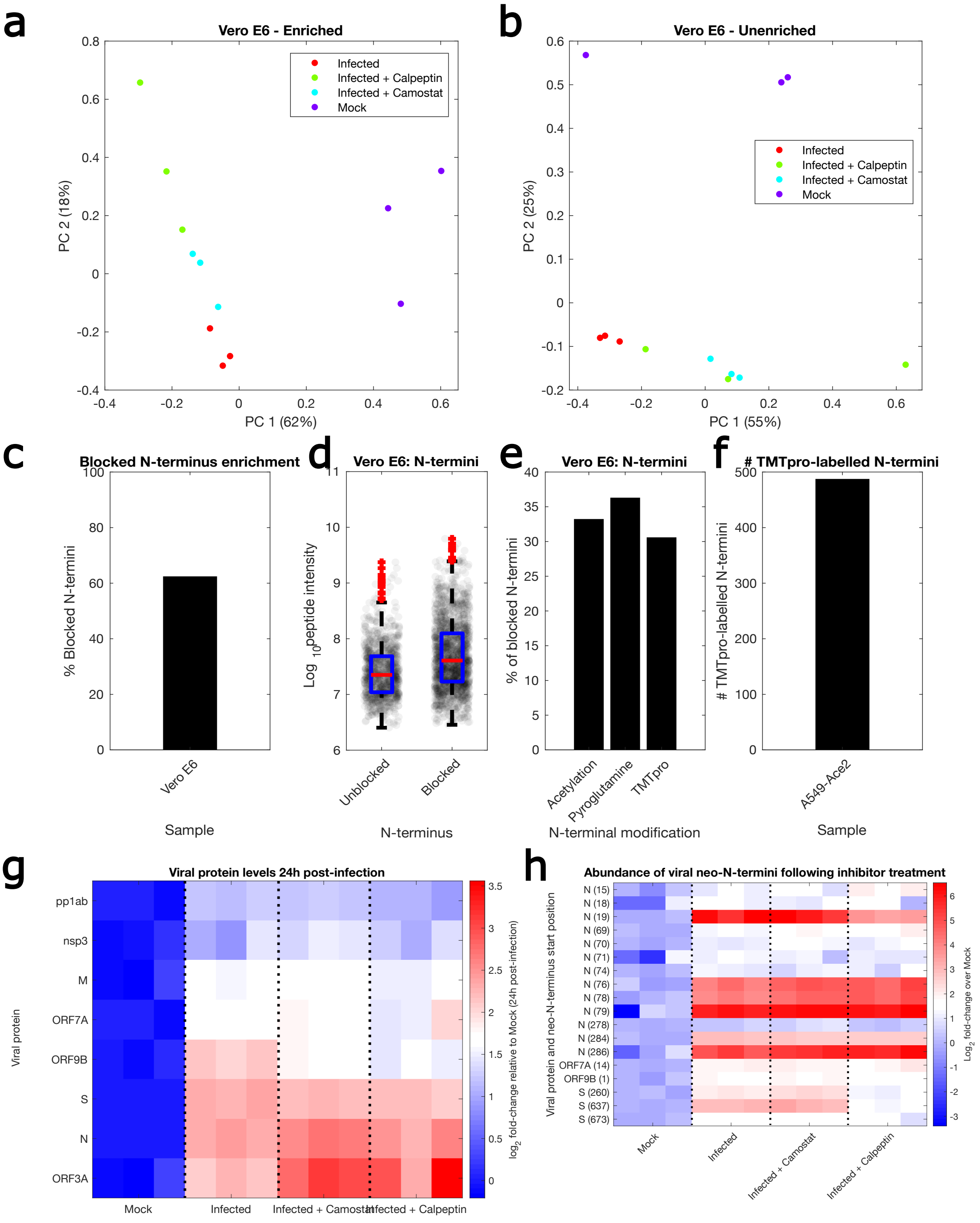

Supplementary Figure 5. Quality control of the protease inhibitor-treated proteomic dataset, both pre- and post-enrichment for N-termini. a-b) Principal component analysis separates infected from mock cells and shows reproducible clustering of biological replicates. c) Enrichment results in a majority of peptide identifications belonging to blocked N-termini, with blocked N-termini more abundant than unblocked N-termini d). Abundance of different blocked N-termini e). f) over 475 TMTpro-labelled N-termini were identified. g) Relative abundance of viral proteins in Vero E6 cells mock- or infected with SARS-CoV-2 and infected in the presence of inhibitors (n = 3biological replicates). h) Relative abundance of neo-N-termini from viral proteins in the same treatment groups. Data is not re-normalised to the total abundance of the viral protein in which the cleavage site is found (n = 3 biological replicates).

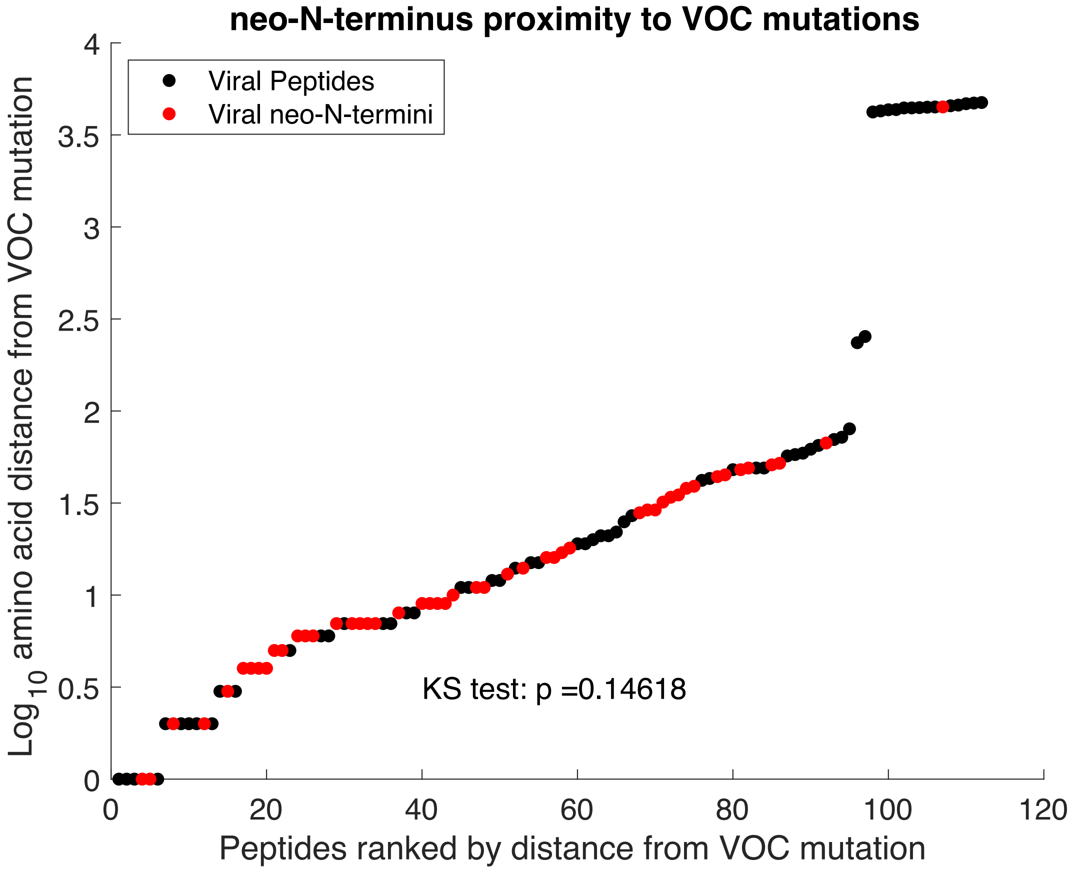

Supplementary Figure 6. Distance of neo-N-termini (red) from mutations present in Variants Of Concern (VOC) relative to all tryptic peptides identified from viral proteins in unenriched datasets (black). p = ns, one-sample Kolmogorov-Smirnov test.

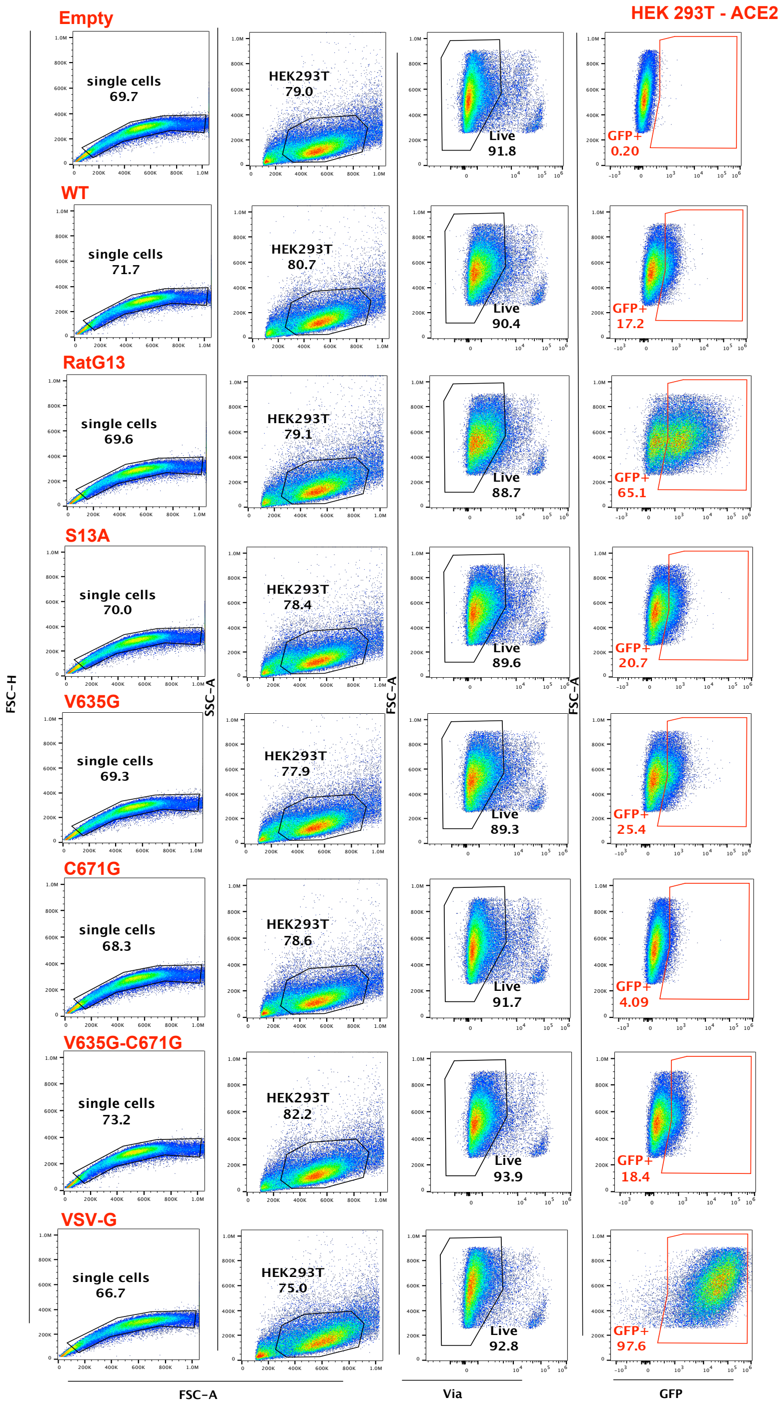

Supplementary Figure 7. Scatter plots illustrating representative data and gating strategy for pseudovirus experiments performed in 293-Ace2 cells. 293-Ace2 cells were infected with spike-pseudotyped lentivectors expressing green fluorescent protein (GFP). Infection was quantified by measuring the percentage of GFP+ cells two days post-infection by flow cytometry. Cells were gated on FSC-H/FSC-A to exclude doublets, and then on SSC-A/FSC-A for size and granularity. Exclusion of dead cells was applied based on labelling for the Via-APC-eFluor780 dye, and infection was then measured based on the percentage of GFP+ cells among live cells.

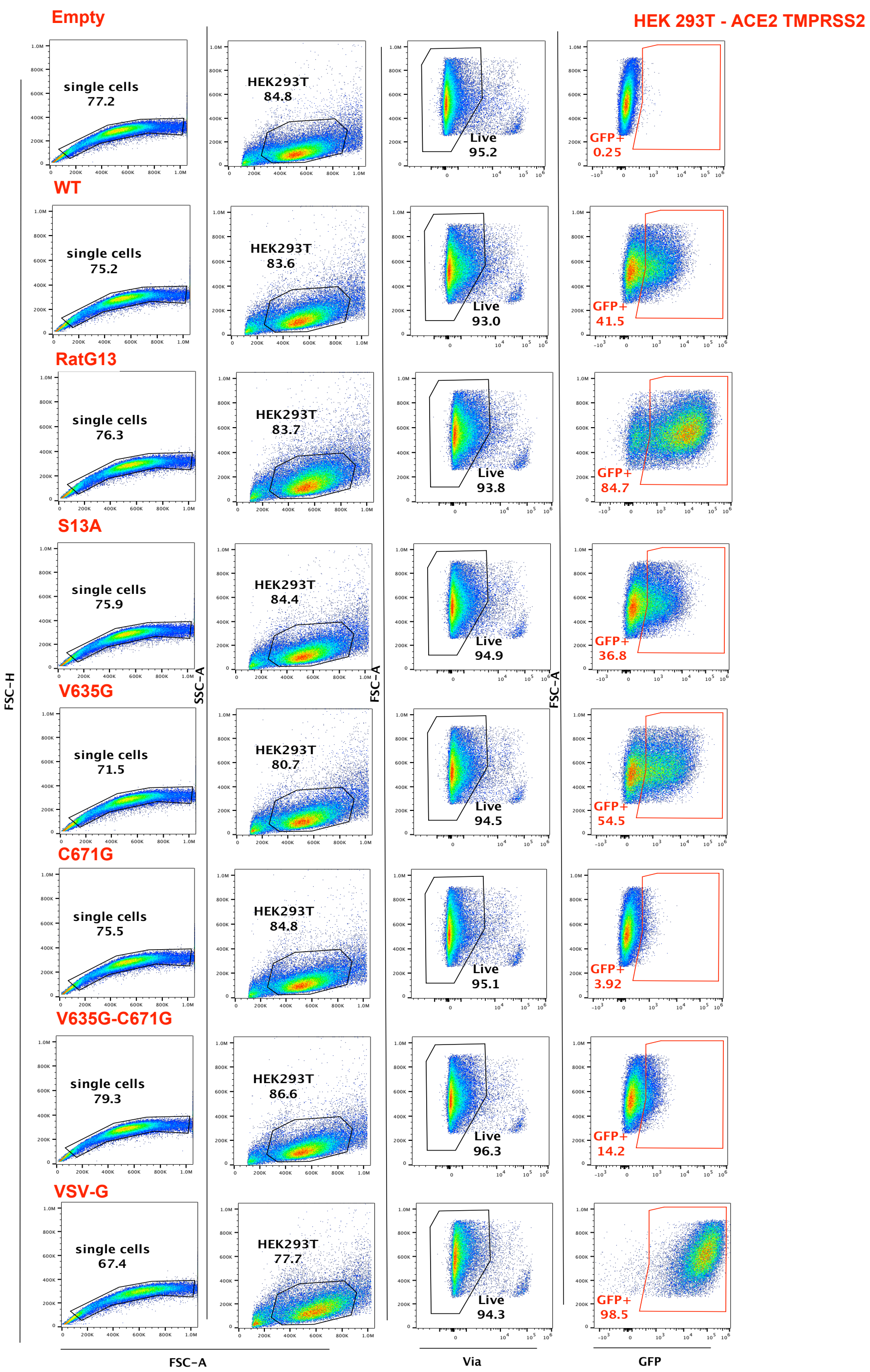

Supplementary Figure 8. Scatter plots illustrating representative data and gating strategy for pseudovirus experiments performed in 293-Ace2-TMPRSS2 cells. 293-Ace2-TMPRSS2 cells were infected with spike-pseudotyped lentivectors expressing green fluorescent protein (GFP). Infection was quantified by measuring the percentage of GFP+ cells two days post-infection by flow cytometry. Cells were gated on FSC-H/FSC-A to exclude doublets, and then on SSC-A/FSC-A for size and granularity. Exclusion of dead cells was applied based on labelling for the Via-APC-eFluor780 dye, and infection was then measured based on the percentage of GFP+ cells among live cells.

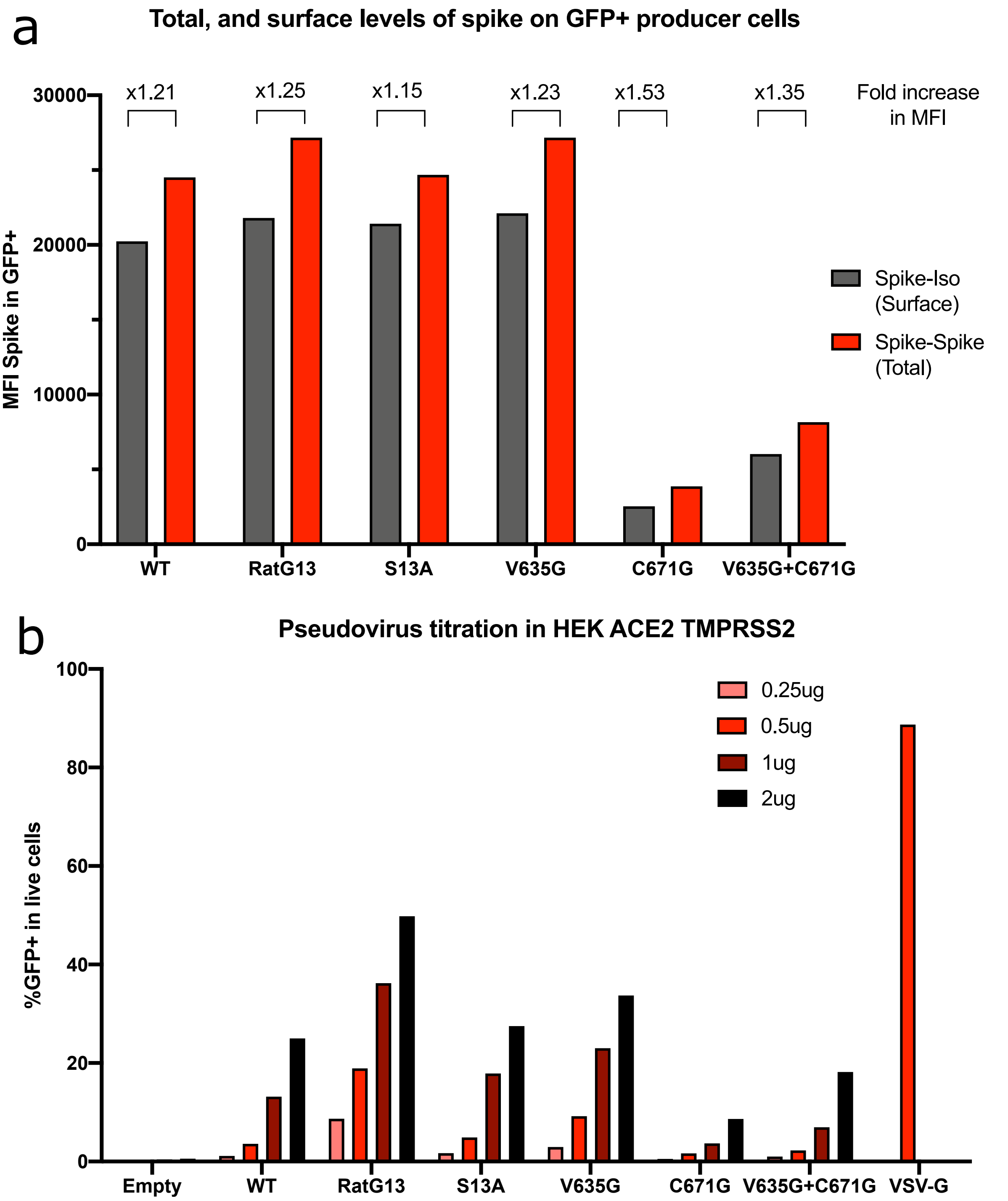

Supplementary Figure 9. Extended characterisation of spike pseudotyped lentivectors. a) Mean fluorescence intensity (MFI) plot examining levels of spike on the surface (spike-isotype control) and total levels of spike (surface and intracellular staining, spike-spike) in transfected (GFP+) HEK 293Tn producer cells (n = 1 biological replicate). Results indicate spike C671G single or V635G/C671G double mutants are present at lower levels in total and at the cell surface, suggesting a defect in stability rather than trafficking to the cell surface. Numbers over columns indicate the fold increase in mean fluorescence intensity of total as compared to surface spike. b) Flow cytometry analysis of HEK-ACE2-TMPRSS2 cells transduced with a dilution series of the different spike pseudotyped lentivectors confirms the observations in Figure 3 remain consistent when input virus is scaled (n = 1 biological replicate). The amount of lentivector used is reported in p24 Gag protein equivalent (legend). The percentage of transduced cells (GFP+) is used to monitor infectivity.

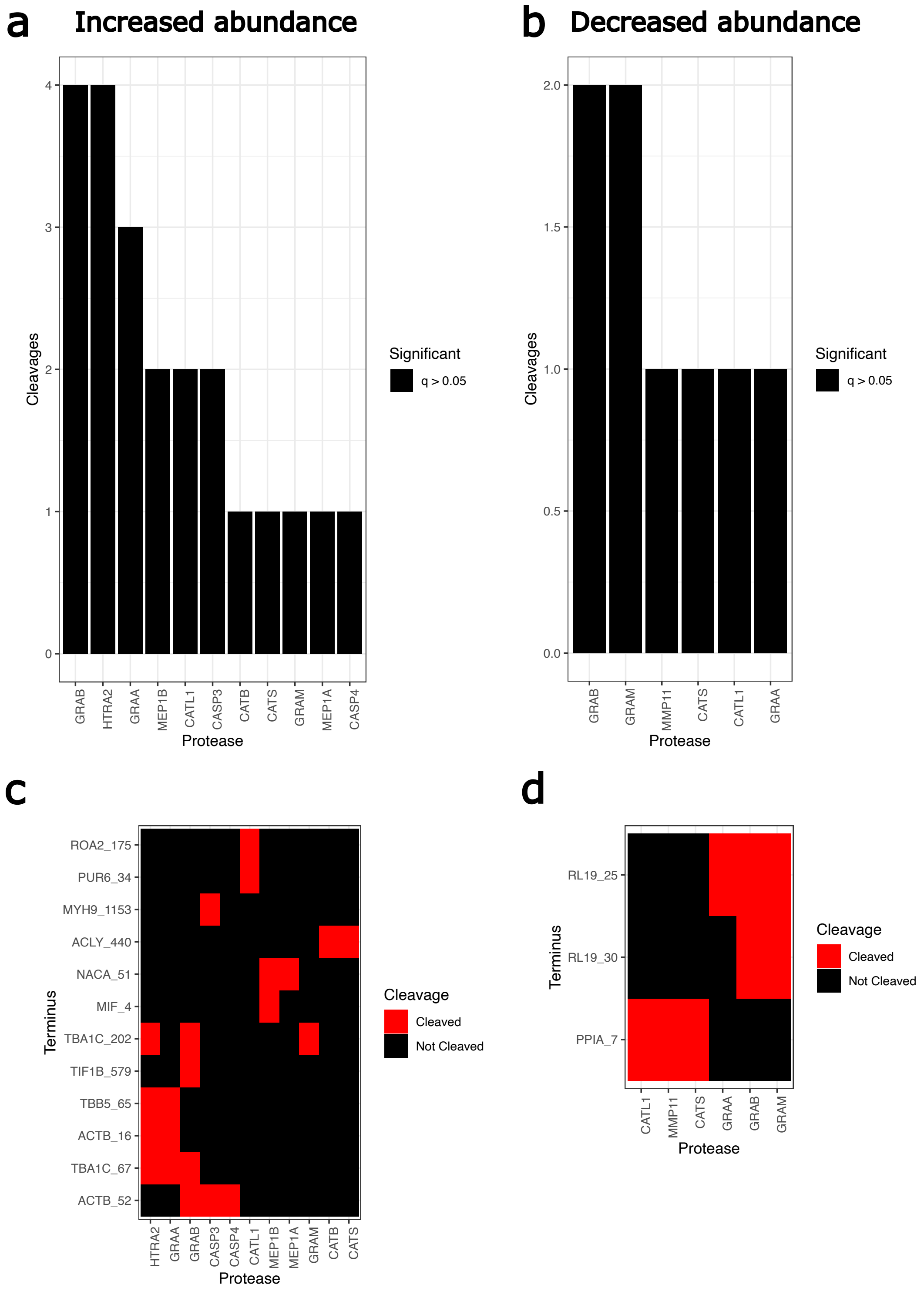

Supplementary Figure 10. TopFIND analysis of cellular neo-N-termini significantly increased or decreased at 24h post-infection relative to mock (n = 3 biological replicates, Linma, Storeys Q-value <= 0.05). a) and b) show the number of neo-N-termini in each dataset associated with each causal protease. c) and d) show heatmaps matching individual neo-N-termini to proteases. Overall, TopFIND did not identify overrepresentation of substrates with these individual proteases (p <= 0.05, Adjusted Fisher's exact test).

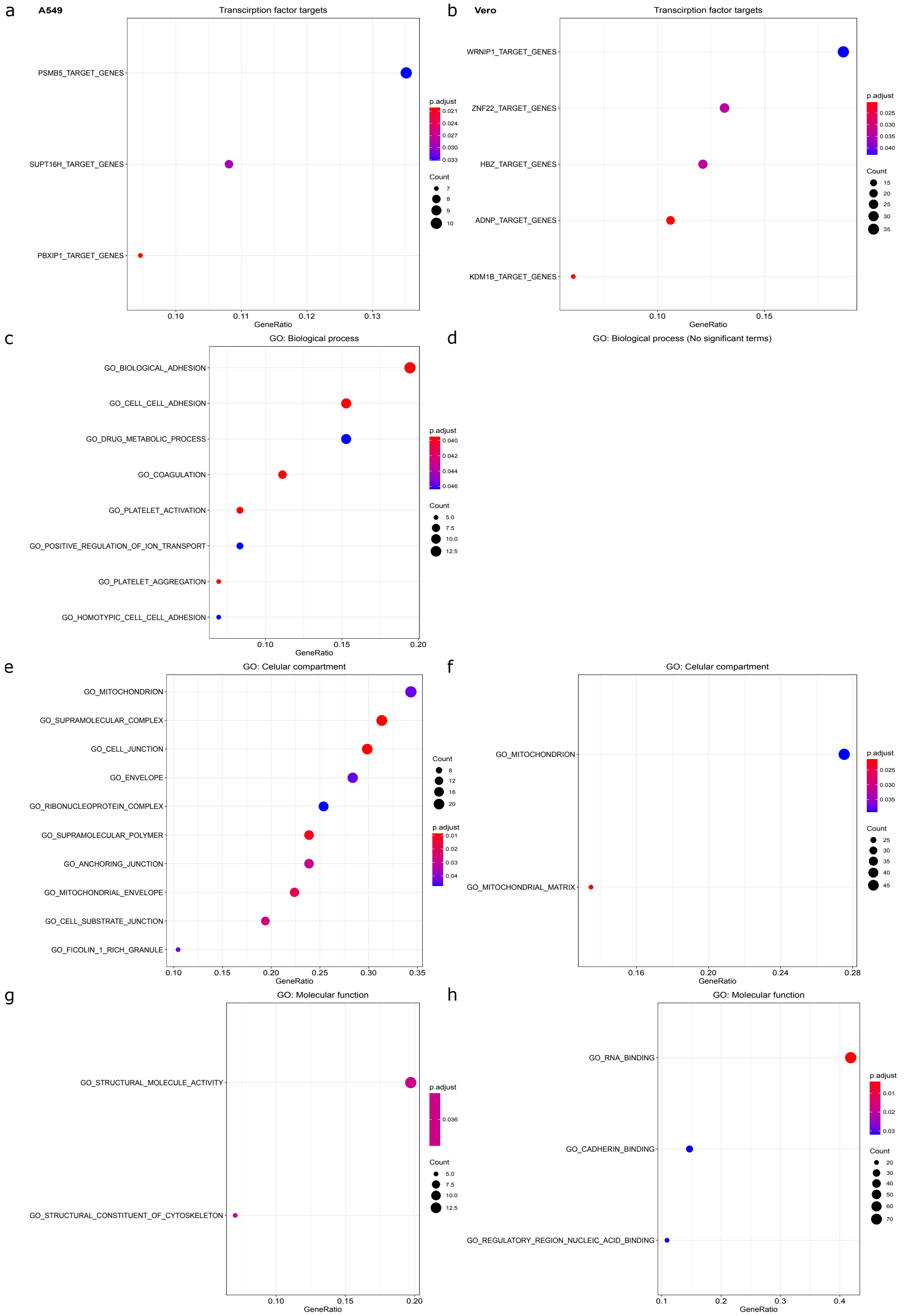

Supplementary Figure 11. Differentially-expressed (Linma, Storeys Q-value adjusted P<=0.05) neo-N-termini from A549-Ace2 and Vero E6 cells at 24h post-infection compared to mock were analysed to determine functional enrichment. Here we show significantly-enriched a/b) transcription-factor targets , as well as gene ontology enrichment for c/d) Biological Processes (note: no significant biological processes identified for Vero E6 cells), e/f) Cellular compartments g/h) Molecular functions.

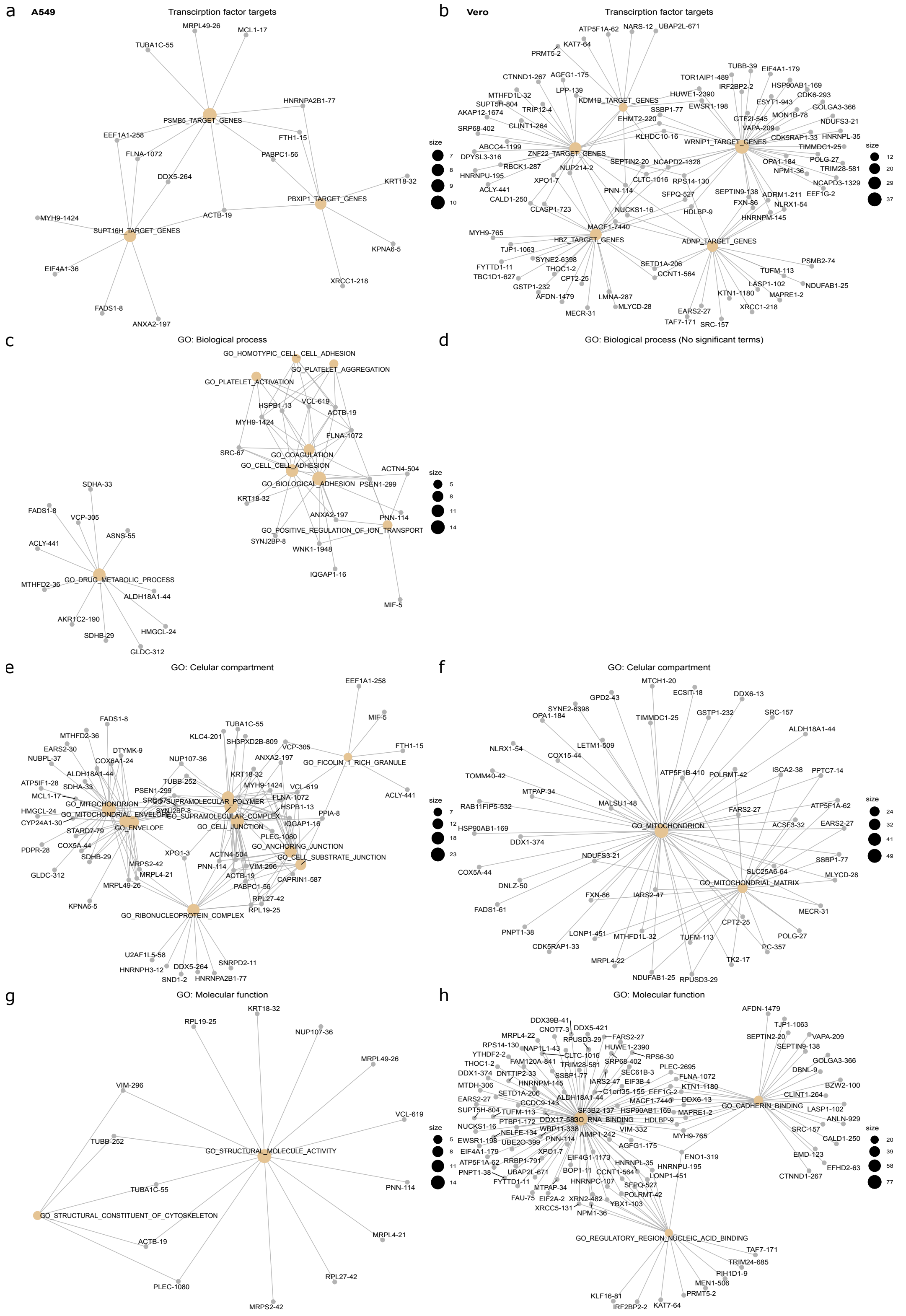

Supplementary Figure 12. Differentially-expressed (Linma, Storeys Q-value adjusted P<=0.05) neo-N-termini from A549-Ace2 and Vero E6 cells at 24h post-infection compared to mock were analysed to determine functional enrichment. Here we highlight the networks of neo-N-termini associated with enriched a/b) transcription factor targets , as well as gene ontology enrichment for c/d) Biological Processes (note: no significant biological processes identified for Vero E6 cells), e/f) Cellular compartments g/h) Molecular. The neo-N-termini are labelled as gene name_cleavage site position.

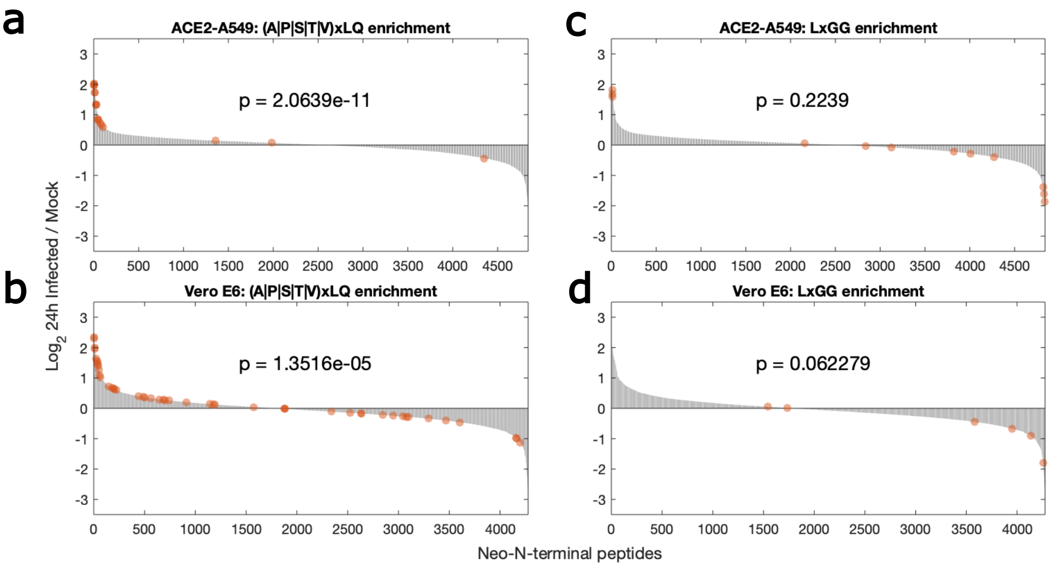

Supplementary Figure 13. a) and b) Distribution of neo-N-termini consistent with the Mpro consensus motif in A549-Ace2 and Vero E6 cells respectively. c) and d) Distribution of neo-N-termini consistent with the PLP consensus motif in A549-Ace2 and Vero E6 cells respectively. Distributions cover all three biological replicates. Enrichment was determined by two-tailed Kolmogorov-Smirnov test.

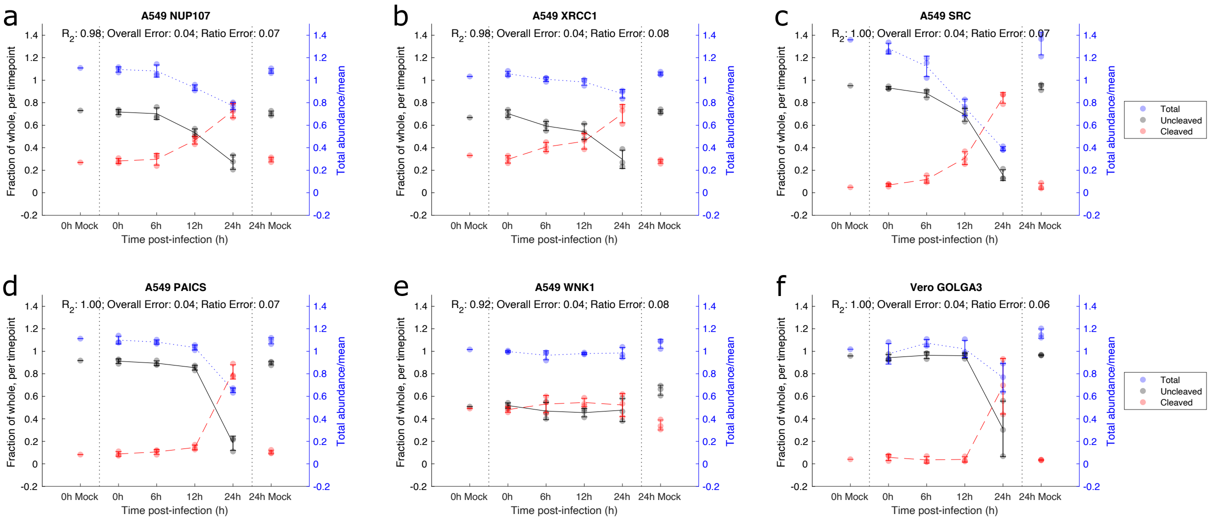

Supplementary Figure 14. Cleavage stoichiometry for cellular proteins cleaved during viral infection was inferred using the HIquant approach (Malioutov et al. 2019). The abundance of cleaved and uncleaved forms of each substrate are calculated on a per-timepoint basis. The total abundance (blue, right axis) is calculated by dividing the total abundance (cleaved and uncleaved) of protein at that timepoint by the mean total abundance across all timepoints). n = 3 biological replicates. Error bars show standard deviation.

+ PLP (Nsp3)

SRC 60 30 15 7.5 3.75 1.87 0.94 µM

kDa

80

Uncleaved SRC

65

50

Cleaved SRC

30

Supplementary Figure 15. In vitro-translated N-terminally GFP-tagged SRC incubated with the indicated concentrations of SARS-CoV-2 PLP shows dose-dependent cleavage of SRC by PLP (n = 3).

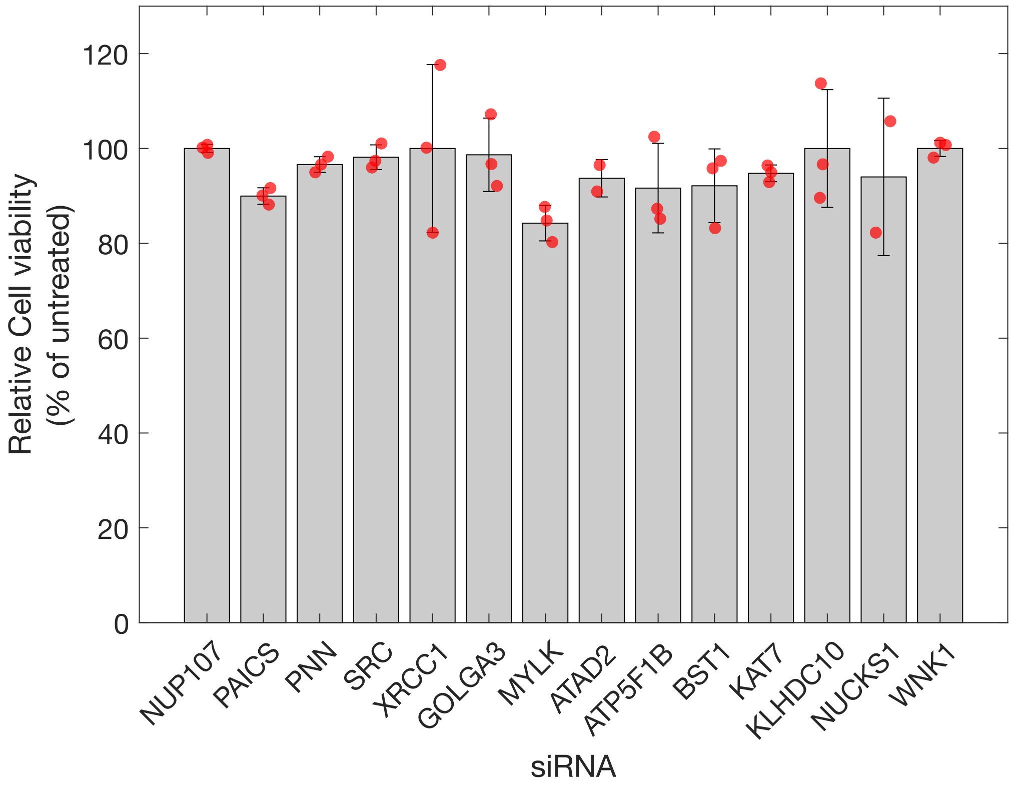

Supplementary Figure 16. Cell viability of siRNA-treated A549-Ace2 cells. Cell viability was assessed by alamar Blue staining and compared to untreated control cells, and a 20% ethanol-lysed control. Error bars represent standard deviation from 3 biological replicates. Red markers indicate individual datapoints.

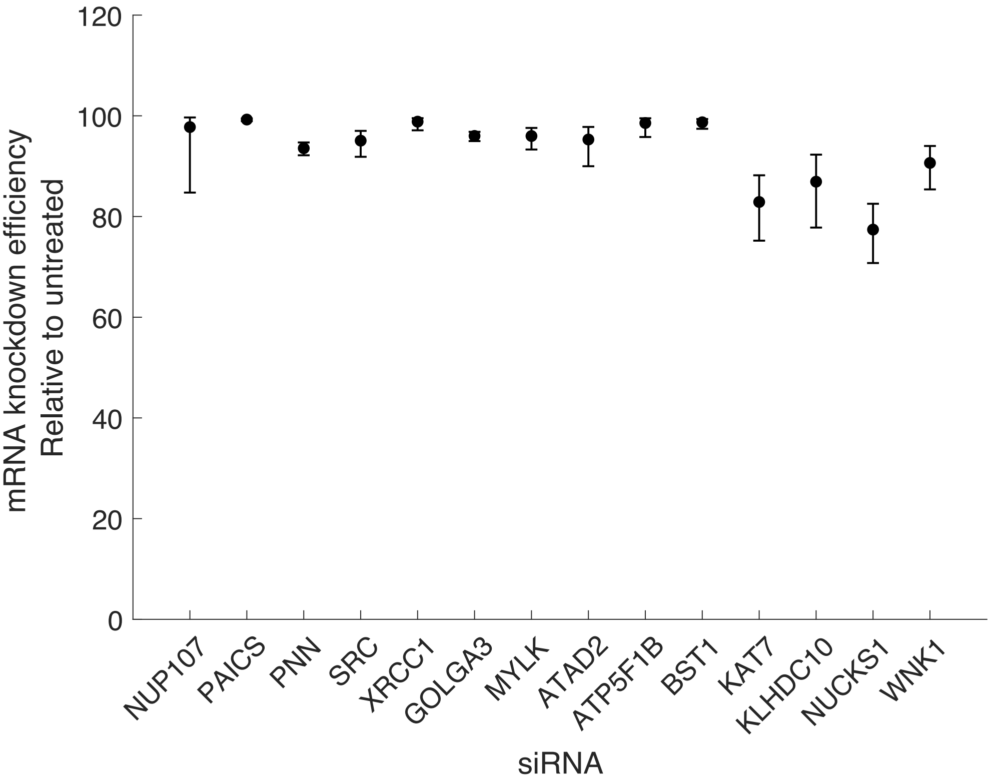

Supplementary Figure 17. mRNA knockdown efficiency measured at 24h post-transfection for SARS-CoV-2 protease substrates in siRNA-treated A549-Ace2 cells. Knockdown efficiency was calculated by qRT-PCR compared to an untreated control by the$2^{-\Delta\Delta Ct}$ method. Error bars represent standard deviation from a minimum of 3 biological replicates.

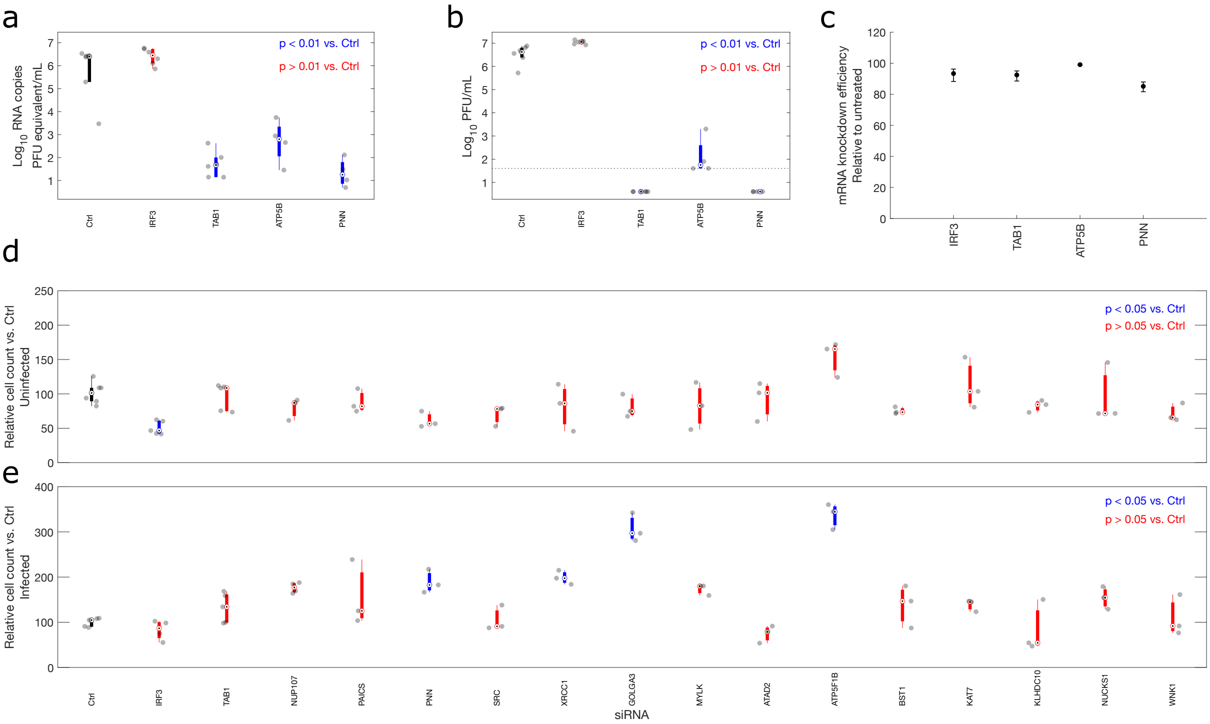

Supplementary Figure 18. siRNA validation on previously identified SARS-CoV-2 protease substrates, including the antiviral substrate IRF3. a) SARS-CoV-2 RNA copies, b) Viral titres (PFU = plaque forming units). n = 6 biologically independent samples for Ctrl and IRF3/TAB1, otherwise n = 4 biologically independent samples. c) KO efficiency relative to a scrambled siRNA control calculated by the$2^{-\Delta\Delta Ct}$ method calculated at 24h post-transfection. n = 6 (IRF3, treated) and 5 ((TAB1, treated) all others n = 4 biologically independent samples. Relative cell numbers as determined by counting Hoechst 33258-stained nuclei in d) Mock and e) Infected cells, 72h post-infection/mock infection. n = 5 biologically independent samples (Ctrl, IRF3, TAB1) or n = 4 biologically independent samples (all other samples). Boxplot minima/maxima represent the furthest non-outlier datapoints, centre the median, and bounds of box the interquartile range. Outliers are defined as datapoints >1.5 times the interquartile range from the bottom or top of the box. n≥3 biological replicates. In panel C, the datapoints represent mean+/- standard deviation. Significance was determined by One-way ANOVA, using Tukey's correction for multiple comparisons.

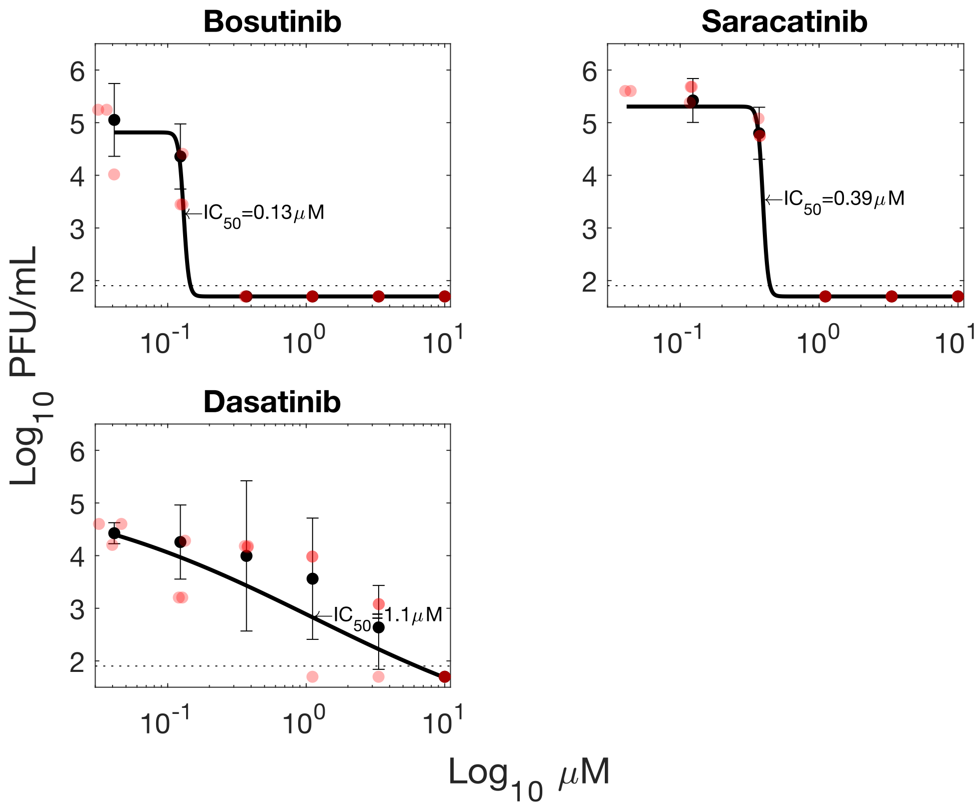

Supplementary Figure 19. Additional Inhibitors targeting SRC kinase reduce SARS-CoV-2 titres in A549-Ace2 cells. Line represents best fit. Black circles and error bars represent mean and standard deviation. n = 3 biologically independent samples. Red circles indicate individual datapoints. The limit of detection in the plaque assay was calculated to be 40 PFU/mL (dotted line). PFU = plaque-forming units.

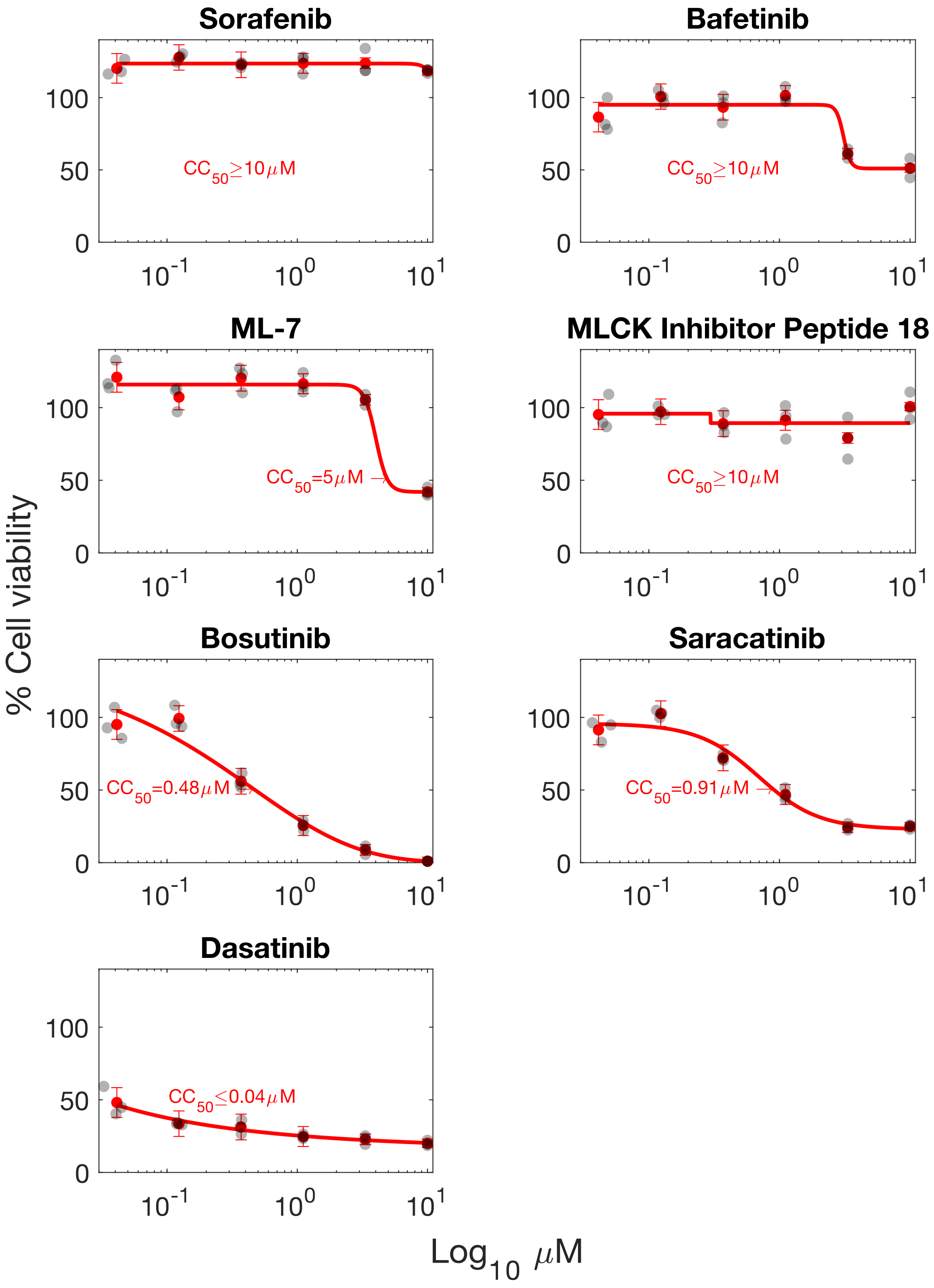

Supplementary Figure 20. Cell viability and CC_{50} calculations for inhibitor-treated A549-Ace2 cells. Cell viability was assessed by Celltiter Glo staining and compared to untreated control cells, and a 20% ethanol-lysed control. Line represents best fit. Red circles and error bars represent mean and standard deviation. n = 3 biologically independent samples. Grey circles indicate individual datapoints.
