# Supplementary material for: Characterising proteolysis during SARS-CoV-2 infection identifies viral cleavage sites and cellular targets with therapeutic potential
Source: Nat Commun. 2021 Sep 21;12:5553. doi: 10.1038/s41467-021-25796-w (PMC8455558; doi:10.1038/s41467-021-25796-w)
Supplement: Supplementary file 3 — Description of Additional Supplementary Information [file 41467_2021_25796_MOESM3_ESM.docx]

Supplementary Data 1. Viral neo-N-termini identified from SARS-CoV-2-infected A549-Ace2 cells - .csv

Supplementary Data 2. Viral neo-N-termini identified from SARS-CoV-2-infected Vero E6 cells - .csv

Supplementary Data 3. All Viral peptides identified accross enriched and unenriched A549-Ace2 and Vero E6 datasets - .csv

Supplementary Data 4. Quantification data for all N- and neo-N-termini quantified from SARS-CoV-2 infected A549-Ace2 cells - .csv

Supplementary Data 5. Quantification data for all N- and neo-N-termini quantified from SARS-CoV-2 infected Vero E6 cells - .csv

Supplementary Data 6. Viral neo-N-termini from A549-Ace2 cells located within 5 amino acids of characteristic mutations from Variants of Interest or Variants of Concern circulating May2021 - .csv

Supplementary Data 7. Viral neo-N-termini from A549-Ace2 cells located within 10 amino acids of characteristic mutations from Variants of Interest or Variants of Concern circulating May2021 - .csv

Supplementary Data 8. TopFIND analysis of neo-N-termini significantly increased or decreased in abundance in A549-Ace2 cells (24h Infected/24h Mock) - .xlsx

Supplementary Data 9. Oligo sequences and reagent details - .csv
