# Supplementary material for: Characterising proteolysis during SARS-CoV-2 infection identifies viral cleavage sites and cellular targets with therapeutic potential
Source: Nat Commun. 2021 Sep 21;12:5553. doi: 10.1038/s41467-021-25796-w (PMC8455558; doi:10.1038/s41467-021-25796-w)
Supplement: Supplementary file 13 — Reporting Summary [file 41467_2021_25796_MOESM13_ESM.pdf]

nature portfolio

Corresponding author(s): Edward Emmott
Last updated by author(s): Aug 4, 2021

Reporting Summary

Nature Portfolio wishes to improve the reproducibility of the work that we publish. This form provides structure for consistency and transparency in reporting. For further information on Nature Portfolio policies, see our Editorial Policies and the Editorial Policy Checklist.

Statistics

For all statistical analyses, confirm that the following items are present in the figure legend, table legend, main text, or Methods section.

n/a	Confirmed	
☐	☒	The exact sample size (*n*) for each experimental group/condition, given as a discrete number and unit of measurement
☐	☒	A statement on whether measurements were taken from distinct samples or whether the same sample was measured repeatedly
☐	☒	The statistical test(s) used AND whether they are one- or two-sided<br>*Only common tests should be described solely by name; describe more complex techniques in the Methods section.*
☒	☐	A description of all covariates tested
☐	☒	A description of any assumptions or corrections, such as tests of normality and adjustment for multiple comparisons
☐	☒	A full description of the statistical parameters including central tendency (e.g. means) or other basic estimates (e.g. regression coefficient) AND variation (e.g. standard deviation) or associated estimates of uncertainty (e.g. confidence intervals)
☐	☒	For null hypothesis testing, the test statistic (e.g. *F*, *t*, *r*) with confidence intervals, effect sizes, degrees of freedom and *P* value noted<br>*Give P values as exact values whenever suitable.*
☒	☐	For Bayesian analysis, information on the choice of priors and Markov chain Monte Carlo settings
☒	☐	For hierarchical and complex designs, identification of the appropriate level for tests and full reporting of outcomes
☒	☐	Estimates of effect sizes (e.g. Cohen's *d*, Pearson's *r*), indicating how they were calculated

Our web collection on statistics for biologists contains articles on many of the points above.

Software and code

Policy information about availability of computer code

Data collection	Maxquant (v1.6.7.0) was used for initial proteomic data analysis.
Data analysis	Custom code written in Matlab R2019b as well as R (v4.0.3) scripts were used for downstream data analysis. All this code has been deposited at https://github.com/emmottlab/sars2nterm where it is freely accessible and has all the necessary supporting raw data for independent reproduction of the data analysis and figures in this manuscript. Further software used includes Graphpad Prism 8, and FlowJo v10.7.1.

For manuscripts utilizing custom algorithms or software that are central to the research but not yet described in published literature, software must be made available to editors and reviewers. We strongly encourage code deposition in a community repository (e.g. GitHub). See the Nature Portfolio guidelines for submitting code & software for further information.

Data

Policy information about availability of data

All manuscripts must include a data availability statement. This statement should provide the following information, where applicable:
- Accession codes, unique identifiers, or web links for publicly available datasets
- A description of any restrictions on data availability
- For clinical datasets or third party data, please ensure that the statement adheres to our policy

The proteomics data generated in this study have been deposited in the ProteomeXchange Consortium (http://proteomecentral.proteomexchange. org) via the PRIDE repository (77). Specifically the A549-Ace2 datasets can be found under PRIDE accession numbers PXD021145 [https://www.ebi.ac.uk/pride/archive/projects/PXD021145], PXD021152 [https://www.ebi.ac.uk/pride/archive/projects/PXD021152] and PXD021402 [https://www.ebi.ac.uk/pride/archive/projects/PXD021402], Vero E6 datasets under PXD021154 [https://www.ebi.ac.uk/pride/archive/projects/PXD021154], PXD021153 [https://www.ebi.ac.uk/pride/archive/projects/PXD021153] and PXD021403 [https://www.ebi.ac.uk/pride/archive/projects/PXD021403], and Protease Inhibitor experiments under PXD023539 [https://

www.ebi.ac.uk/pride/archive/projects/PXD023539], PXD023538 [https://www.ebi.ac.uk/pride/archive/projects/PXD023538] and PXD023540 [https://www.ebi.ac.uk/pride/archive/projects/PXD023540].
Raw images corresponding to cell viability have been deposited in the Zenodo repository under DOI: 10.5281/zenodo.4984272 [http://doi.org/10.5281/zenodo.4984272].

Other reagent and oligo sequence details are described in Supplementary table 9. Source data are provided with this paper.
Raw data for cell viability, qRT-PCR and viral titres as well as the code for analysis and statistics is included at https://github.com/emmottlab/sar2nterm, and is also included within the sourceData.zip.

PDB 6X6P is referenced in this study, and is available through the protein data bank.

Field-specific reporting

Please select the one below that is the best fit for your research. If you are not sure, read the appropriate sections before making your selection.

☒ Life sciences ☐ Behavioural & social sciences ☐ Ecological, evolutionary & environmental sciences

For a reference copy of the document with all sections, see nature.com/documents/nr-reporting-summary-flat.pdf

Life sciences study design

All studies must disclose on these points even when the disclosure is negative.

Sample size	It is accepted practice in the proteomics field that biological triplicates are sufficient for measuring high-confidence proteomic changes in this type of study. Sample size was therefore three biological replicates. Three biological replicates were all employed for other assays except where indicated. Recent published examples from the proteomics literature include Gordon et al. A SARS-CoV-2 protein interaction map reveals targets for drug repurposing. Nature, 2020
Data exclusions	On cell viability assays performed at the Institut Pasteur, replicates were excluded if they were considered outliers (if cell viability was significantly different compared to other replicates). A small number of qRT-PCR datapoints were excluded where no Ct was measure (i.e. the reaction failed). No other data were excluded from the study.
Replication	Proteomics data were analysed at Liverpool, UK, and validated in the manuscript by orthogonal methods (siRNA screens, inhibitor screens, in vitro, cell-based assays) in collaborators labs based in France, the USA and Australia. Experiments are contained within individual TMTpro experiments, allowing assessment of biological variability, while minimising the impact of technical variation. The othogonal assays used to validate the proteomics data were successful.
Randomization	Samples were assigned to randomised TMTpro labels for sample processing using the Matlab 'randperm' function.
Blinding	The corresponding author was responsible for proteomic sample processing and data analysis so blinding was not possible. However, as is standard practice in proteomics data were acquired and analyzed systematically using established scoring criteria. All files necessary to reproduce the figures and analysis pipeline are included in the PRIDE upload (mqpar file to reproduce Maxquant analysis), and github (annotated custom code takes Maxquant output files to generate the paper figures). Study design enabled all mock and infected samples within a dataset to be process simultaneously, minimizing batch effects. Inhibitor and siRNA studies were conducted by a collaborator and were blinded.

Reporting for specific materials, systems and methods

We require information from authors about some types of materials, experimental systems and methods used in many studies. Here, indicate whether each material, system or method listed is relevant to your study. If you are not sure if a list item applies to your research, read the appropriate section before selecting a response.

Materials & experimental systems

n/a	Involved in the study	
☐	☒	Antibodies
☐	☒	Eukaryotic cell lines
☒	☐	Palaeontology and archaeology
☒	☐	Animals and other organisms
☒	☐	Human research participants
☒	☐	Clinical data
☒	☐	Dual use research of concern

Methods

n/a	Involved in the study	
☒	☐	ChIP-seq
☐	☐	Flow cytometry
☒	☐	MRI-based neuroimaging

Antibodies

Antibodies used	human anti-spike mAb 48 Donated by H. Mouquet N/A<br>the mouse anti-p24 Gag R&D Systems MAB7360

	Goat anti-Human IgG Fc fragment, DyLight 800-conjugated Bethyl Laboratories A80-304D8<br>Goat anti-Mouse IgG (H+L), DyLight 680-conjugated ThermoFisher Scientific SA5-35518<br>Goat anti-Rabbit IgG, HRP-linked Cell Signaling #7074<br>Rabbit polyclonal anti-ADE2 (PAICS) Bethyl Laboratories A304-547A-T<br>Rabbit polyclonal anti-Golgin-160 (GOLGA3) Bethyl Laboratories A303-404A-T<br>Rabbit monoclonal anti-Beta tubulin Cell Signaling #2128
Validation	Western blotting was employed wither for transfection-based/lentivirus studies, enabling assessment against an untransfected control (anti-p24 Gag, anti-spike), for a commo housekeeping protein (beta-tubulin), of for cleaved cellular proteins, where specific bands corresponding to cleavage products only appeared in the presence of the relevant viral protease. Example blots illstrating bands of correct mass were available for all commercial primary antibodies. The custom anti-spike mAb 48 identified a band of the correct mass only in cells transfected/transduced with SARS-CoV-2 spike.

Eukaryotic cell lines

Policy information about cell lines

Cell line source(s)	ATCC or named collaborators at the Institut Pasteur, Paris, France. Vero E6 (ATCC), A549-Ace2, HEK-Ace2, HEK-Ace2-TMPRSS2 (Olivier Schwatz, Institut Pasteur), HEK 293T (ATCC).
Authentication	All cell lines were purchased direct from ATCC, or from named collaborators who generated the relevent transgenic cell line (e.g. A549-Ace2). No further authentication was performed.
Mycoplasma contamination	All cell lines were tested for mycoplasma contamination and found negative.
Commonly misidentified lines<br>(See ICLAC register)	No commonly misidentified cell lines were used in this study.

Flow Cytometry

Plots

Confirm that:

☒ The axis labels state the marker and fluorochrome used (e.g. CD4-FITC).

☒ The axis scales are clearly visible. Include numbers along axes only for bottom left plot of group (a 'group' is an analysis of identical markers).

☒ All plots are contour plots with outliers or pseudocolor plots.

☒ A numerical value for number of cells or percentage (with statistics) is provided.

Methodology

Sample preparation	HEK-ACE2 +/- TMPRSS2 cells were harvested at 2 days post-infection, washed with PBS and stained for viability with the Via-APC-eFluor 780 dye for 30 min at 4 degrees C. Samples were then washed twice in PBS, and fixed in 2% paraformaldehyde before acquisition.
Instrument	Data was collected on an Attune NxT flow cytometer (ThermoFisher Scientific)
Software	The FlowJo v10.7.1 software (Becton Dickinson) was used to analyze flow cytometry data.
Cell population abundance	The equivalent of 100,000 plated HEK-ACE2 cells were harvested per sample. After gating, the percentage of GFP+ cells was determined in a minimum of 20,000 live singlet cells.
Gating strategy	HEK-ACE2 cells were infected with spike-pseudotyped lentivectors expressing the green fluorescent protein (GFP). Infection was quantified by measuring the percentage of GFP+ cells two days post-infection by flow cytometry. Cells were gated on FSC-H/FSC-A to exclude doublets, and then on SSC-A/FSC-A for size and granularity. Exclusion of dead cells was applied based on labeling for the Via-APC-eFluor 780 dye (ThermoFisher Scientific), and infection was then measured based on the percentage of GFP+ cells among live cells. The gate for GFP+ cells was defined so that the background obtained with a control lentivector devoid of spike remained below 1%.

☒ Tick this box to confirm that a figure exemplifying the gating strategy is provided in the Supplementary Information.
