# Supplementary material for: Characterising proteolysis during SARS-CoV-2 infection identifies viral cleavage sites and cellular targets with therapeutic potential
Source: Nat Commun. 2021 Sep 21;12:5553. doi: 10.1038/s41467-021-25796-w (PMC8455558; doi:10.1038/s41467-021-25796-w)
Supplement: Supplementary file 14 — Source Data [file 41467_2021_25796_MOESM14_ESM.zip › SourceData/UncroppedGels/Uncroped_WBGels_Fig3E.pptx]

## Slide 1
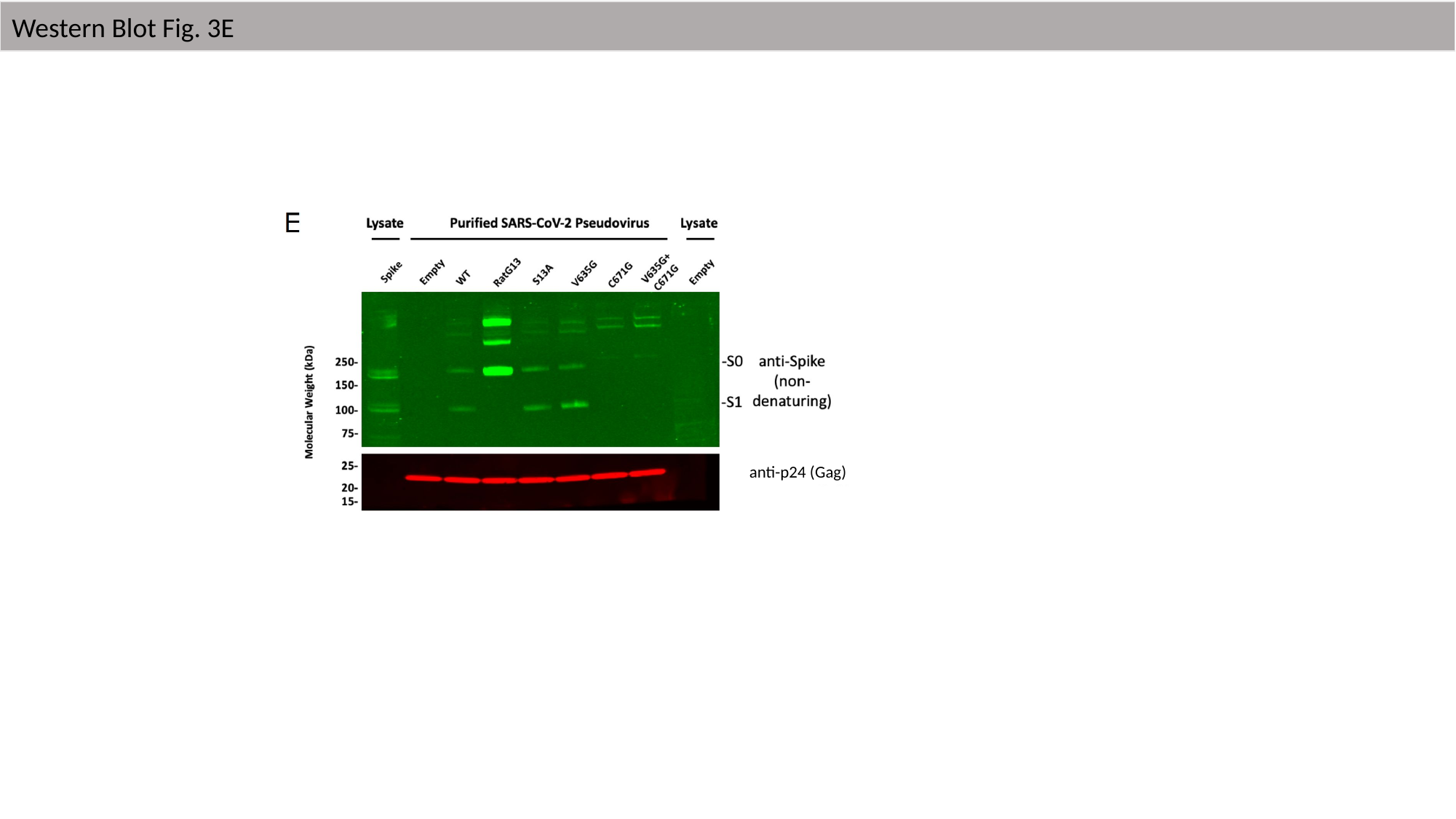

Western Blot Fig. 3E
anti-p24 (Gag)

## Slide 2
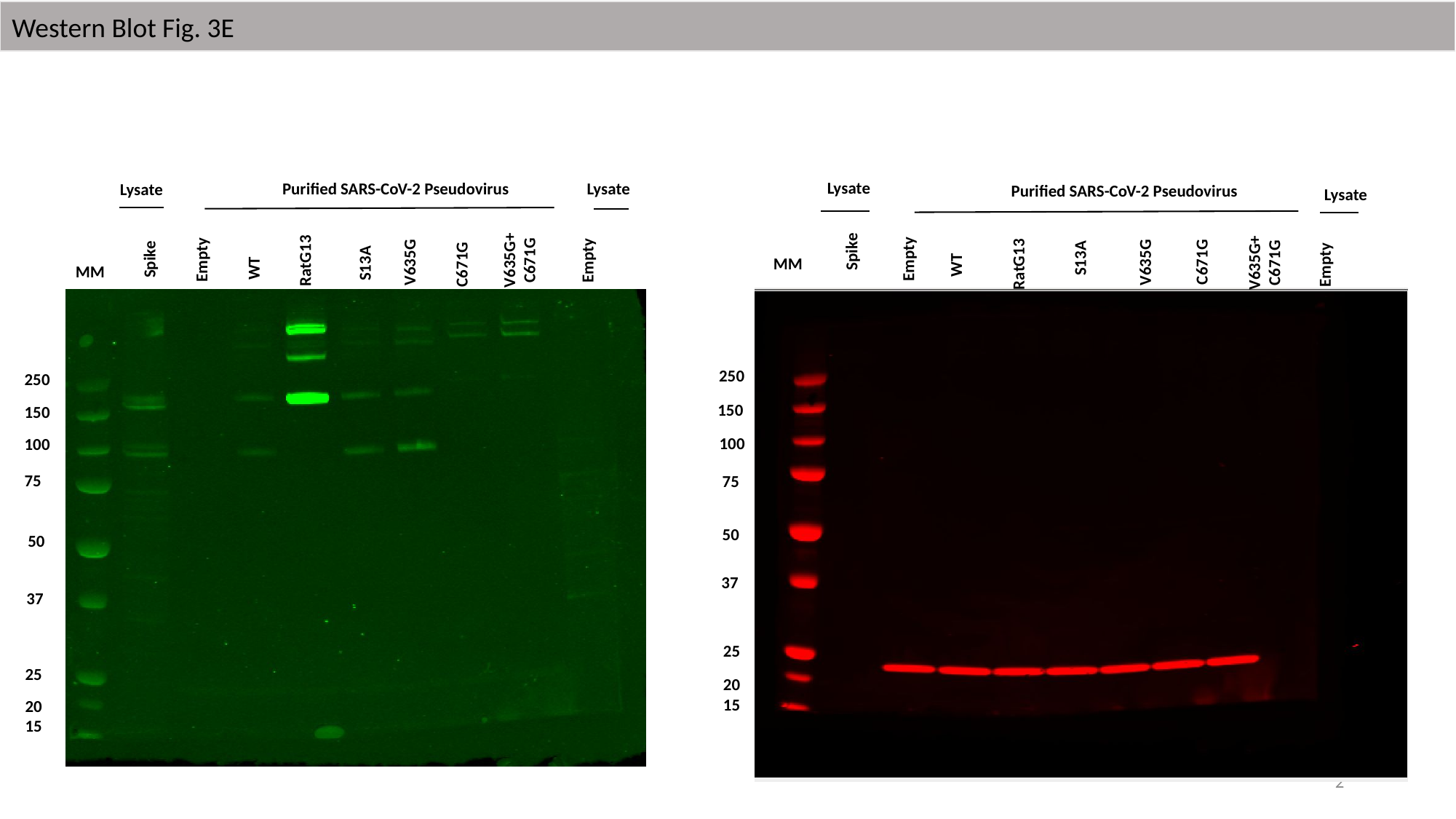

Western Blot Fig. 3E
Lysate
Purified SARS-CoV-2 Pseudovirus
Lysate
Spike
V635G+
C671G
S13A
Empty
MM
C671G
V635G
RatG13
Empty
WT
250
150
100
75
50
37
25
20
15
Lysate
Purified SARS-CoV-2 Pseudovirus
Lysate
V635G+
C671G
Spike
Empty
Empty
RatG13
V635G
S13A
C671G
WT
MM
250
150
100
75
50
37
25
20
15
2
