# Supplementary material for: Characterising proteolysis during SARS-CoV-2 infection identifies viral cleavage sites and cellular targets with therapeutic potential
Source: Nat Commun. 2021 Sep 21;12:5553. doi: 10.1038/s41467-021-25796-w (PMC8455558; doi:10.1038/s41467-021-25796-w)
Supplement: Supplementary file 14 — Source Data [file 41467_2021_25796_MOESM14_ESM.zip › SourceData/UncroppedGels/Uncropped_InVitroTranslationGels_Fig4F_SFig15.pptx]

## Slide 1
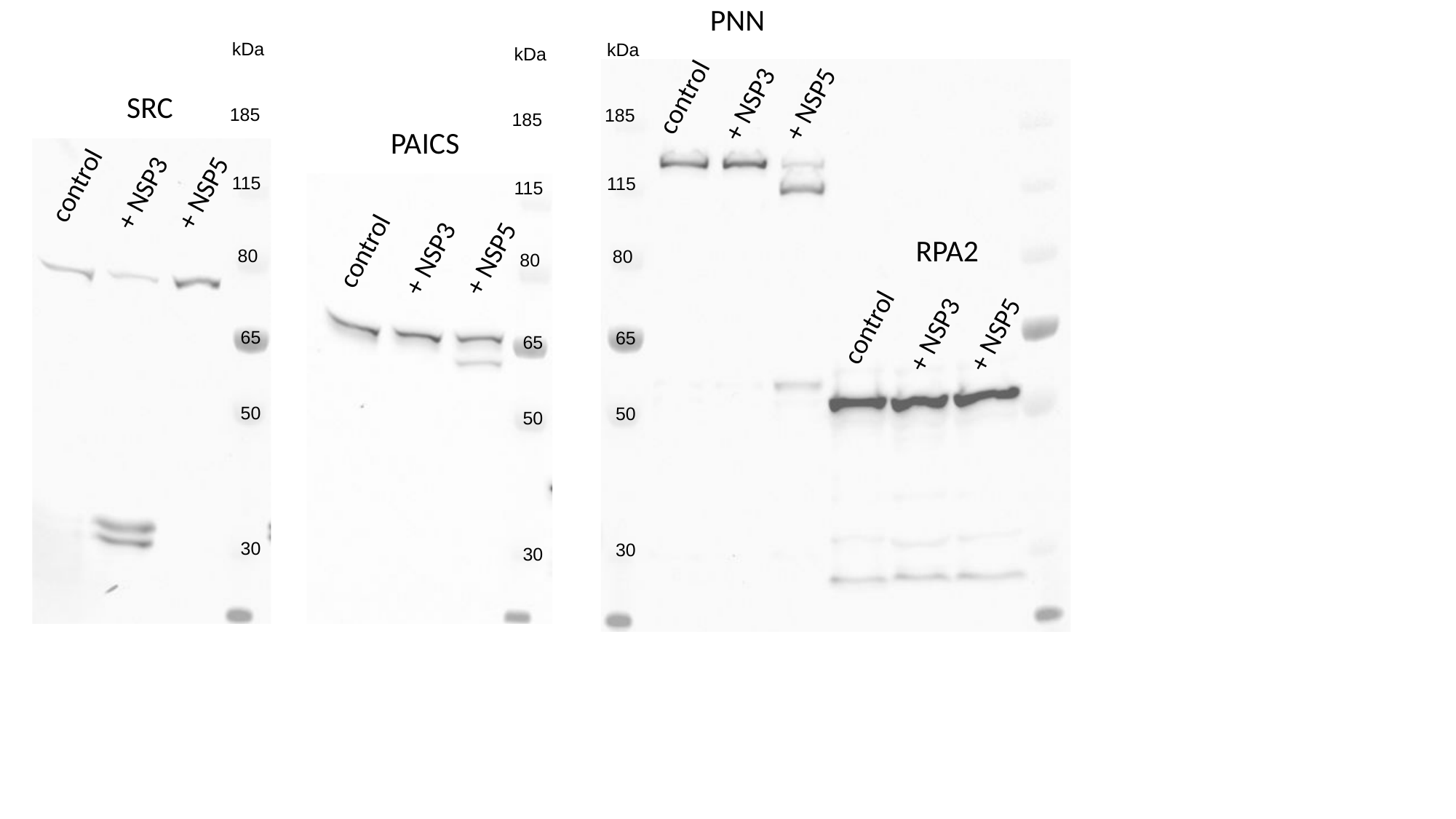

PNN
kDa
kDa
kDa
control
+ NSP5
+ NSP3
SRC
185
185
185
PAICS
control
115
115
+ NSP5
+ NSP3
115
kDa
RPA2
control
+ NSP5
+ NSP3
80
80
80
control
80
+ NSP5
+ NSP3
65
65
65
50
50
50
65
50
30
30
30
30

## Slide 2
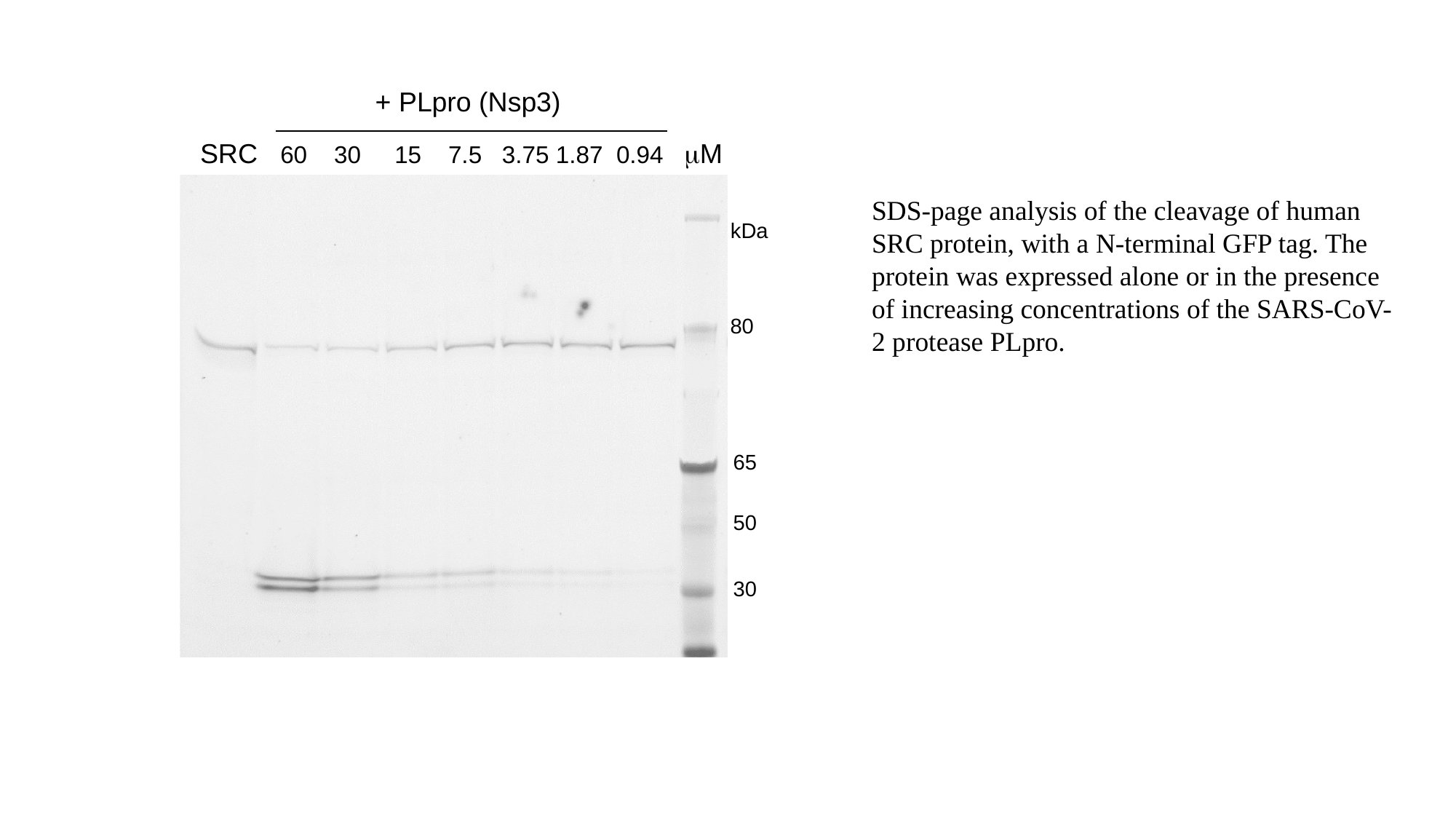

+ PLpro (Nsp3)
SRC 60 30 15 7.5 3.75 1.87 0.94 mM
SDS-page analysis of the cleavage of human SRC protein, with a N-terminal GFP tag. The protein was expressed alone or in the presence of increasing concentrations of the SARS-CoV-2 protease PLpro.
kDa
80
65
50
30
