# Supplementary figures and images for: Characterising proteolysis during SARS-CoV-2 infection identifies viral cleavage sites and cellular targets with therapeutic potential
Source: Nat Commun. 2021 Sep 21;12:5553. doi: 10.1038/s41467-021-25796-w (PMC8455558; doi:10.1038/s41467-021-25796-w)

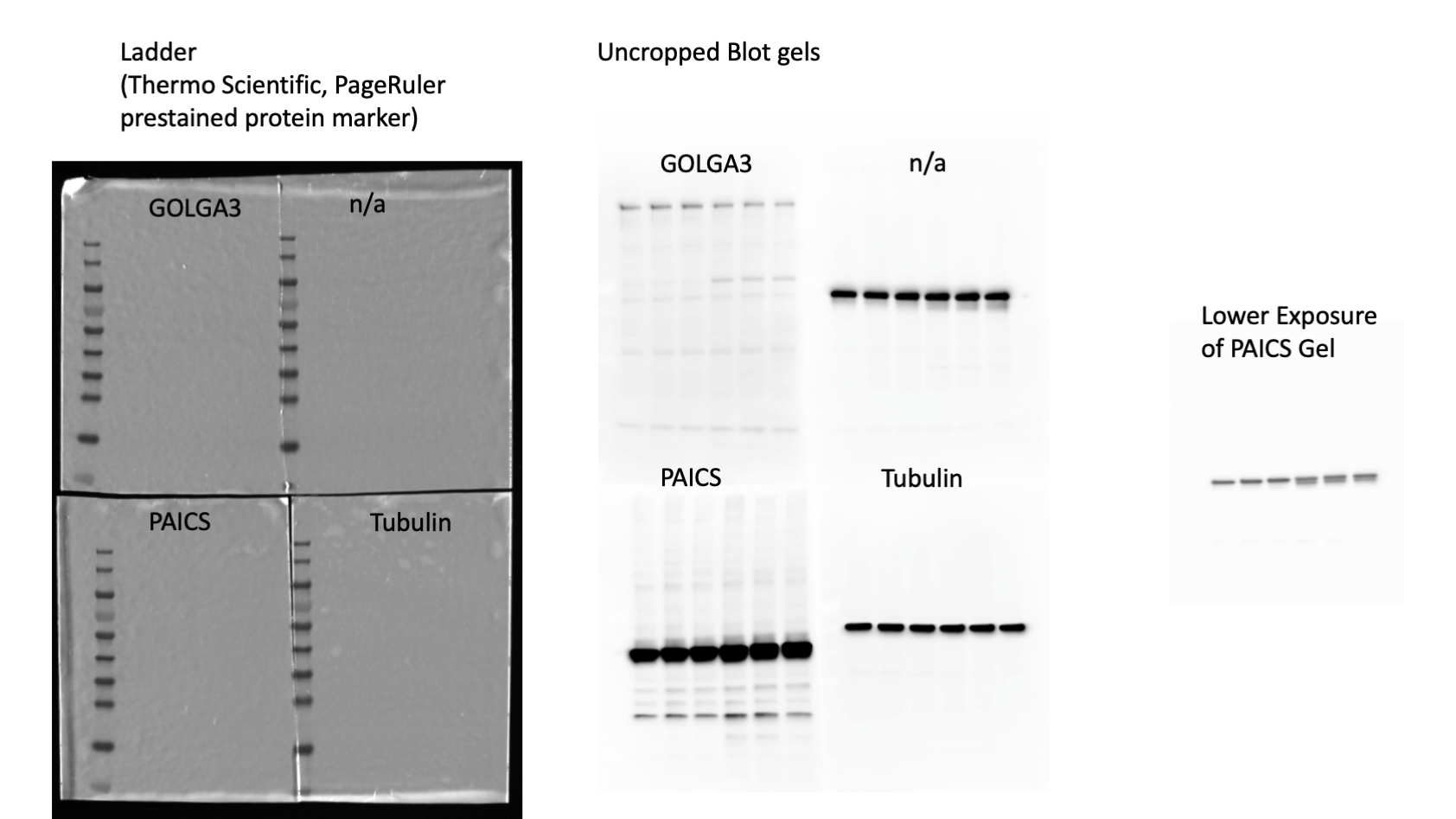

Supplement: Supplementary file 14 — Source Data [file 41467_2021_25796_MOESM14_ESM.zip › SourceData/UncroppedGels/Uncropped_WBgels_Fig4G.png]
