# Supplementary material for: Characterising proteolysis during SARS-CoV-2 infection identifies viral cleavage sites and cellular targets with therapeutic potential
Source: Nat Commun. 2021 Sep 21;12:5553. doi: 10.1038/s41467-021-25796-w (PMC8455558; doi:10.1038/s41467-021-25796-w)
Supplement: Supplementary file 2 — Peer Review File [file 41467_2021_25796_MOESM2_ESM.pdf]

Peer Review File

Reviewer comments, first round -

Reviewer #1 (Remarks to the Author):

Meyer et al. studied the proteolytic cleavage of cellular and viral proteins in vivo upon SARS-CoV-2 infection of A549-ACE2 and Vero E6 cells by using an N-terminomic mass-spectrometry approach. Key findings were validated and inhibitors which target the proteolytically cleaved cellular proteins SRC and MYLK were active against SARS-CoV-2.
Overall, the study represents a rich resource, which adds significant knowledge that can be further explored to identify novel regulatory mechanisms of virus infections and SARS-CoV-2 in particular. A limitation of the study are functional analyses regarding the individual cleavage products identified. The authors use mutational analysis close to the cleavage sites to study S-specific fragments but the resulting data is difficult to interpret. Moreover, I have some concerns regarding the functional analysis after siRNA treatment which would require additional supporting data. I also feel that the use of pleiotropic kinase inhibtors does not help to link the occurrence of cellular protein fragments to pro/antiviral functionality. Still, given the MS dataset and the novelty of the data provided I feel that this manuscript is interesting and should be revised.

Major comments:
1. While the authors provide a detailed analysis of cellular proteins that are potentially cleaved by viral proteases, they barley analyze the global pattern of neo-N-termini of cellular proteins that change upon virus infection. For example, activation of signaling cascades involved in innate immune responses or various stress-activated pathways might also lead to an increased cleavage of specific cellular proteins and hence produces neo-N-termini upon virus infection.
a. The authors should provide a more global analysis of their mass-spectrometry data including, for example pathway enrichment analysis, to potentially identify protease substrates that belong to the same pathway and could thereby indicate viral perturbations on pathway level.
b. Additionally, do the authors detect an overrepresentation of amino acid sequence motives on neo-N-termini that would suggest an activation of specific cellular proteases upon virus infection?

2. Figure 1E, F present one of the most interesting datasets but the data is in my opinion not well presented.
a. mention q-value and fold change cutoffs (dashed lines) in legend
b. What are purple dots? Please clarify
c. please provide an enlarged version of these volcano plots where the most significantly changing neo-N-termini are labeled
d. please provide a scatter plot comparing the log2 fold change of neo-N-termini between A549 and Vero cells and comment on shared and unique neo-N-termini. It would be re-assuring that similar peptides are identified in the individual datasets.

3. The inhibition of proteases followed by N-terminomics is interesting. However, the authors correctly mention in line 375 that lack of TMPRSS2 in standard Vero-cells and likely also in A549-Ace2 cells. Can the authors please provide functional data that TMPRSS2 is active in their cell types? Otherwise the interpretation is very difficult – i.e. it is not poissible to functionally link the inhibitor data to activity of the inhibited proteases and phenotypes observed in the further experiments.

4. The pseudotyping experiment is not well described in text and figure legends and is somehow difficult to interpret. There is a lot of room for interpretation of this result, which – to the credit of the authors – they provide. However, it would be easy to gain further insights and support of these data with moderate effort:
a. Can the authors please provide an exemplary FACS blot comparing the most prominent candidates (e.g. V635G vs wt) (e.g scatter blots or MFI histogram)? This would be very reassuring on the effects shown.
b. It would be interesting to see the expression levels of transfected S mutants in 293T (producer) cells. This could indicate whether the introduced mutations would affect protein expression already in the producer cell or whether there is an issue on trafficking of the protiens. How do the different

mutations localize intracellularly?
c. While lentivector particles can be obtained in the absence of glycoproteins, interpretation of infectivity is depending on the presence of S on the lentivector pseudotypes. If the authors want to make statements on different infectivity (or loading of S on lentivectors) titrations are be required. The authors should use different doses of pseudotyped virus for the assay in order to allow proper appreciation of the effect.

5. I am a bit concerned regarding the siRNA data even though this data is well controlled per se. Despite the surprisingly high knockdown efficiency (particularly considering transient knockdown in A549 cells, which are often not so easy to target) no toxicity of gene depletion is detected. However, some of the candidates have been shown to be essential for growth of eukaryotic cells (e.g. e.g. NUP107, PAICS, PNN and others/ doi:10.1007/s11010-010-0494-6, doi: 10.1186/s40659-018-0172-9, doi: 10.1242/jcs.100859). It is further surprising that all (!) tested candidates have a similar (inhibitory) effect. Even though the results are clear and appear controlled I feel that some additional proof that the effect is not simply due to reduced cell viability would be required. I would, for instance be more convinced if another readout that assures the viability of cells at the end of the experiments (e.g. by imaging and metabolic assays) would be used. It would, for instance be expected that the highly reduced virus load also reduces cell cytotoxicity imposed by the virus in knockdown cells as compared to control conditions.

Minor comments:
• General comments
o Add number and type of replicates for each experiment in figure legend
o Always mention exact type of statistical test (e.g. two-sided unpaired student’s t-test) in text/figure legends
• Even though the authors mostly focus on cleavage products it would be good to also comment whether non-canonical translation start sites could contribute to the observed protein patterns.
• Line 72
o Please use the appropriate gene and (protein) name from uniprot, e.g. Furin (FURIN) and Transmembrane protease serine 2 (TMPRSS2)
• Line 192
o Please specify the exact type of t-test and multiple hypothesis test used
• Figure 1 and 6
o Please unify y-axis label by using PFU/ml in Figure 1 and introduce PFU in the legend
• Figure 1 legend
o Please introduce abbreviations in legend, e.g. bRP Fractionation in Figure 1
• Figure 2B-D
o Please label all neo-N-termini that are mentioned in the text
• Figure 2G
o May rather be provided as supplement figure since it is too technical for figure 2
• Figure 3B
o Right y-axis label formatting error
• Figure 5B scale
o
• Line 800-801
o Remove "…addition of TMPRSS2 was induced by…"
• Line 843
o (REF) missing
• Line 909
o Unit missing for NCE, 30%?

Reviewer #2 (Remarks to the Author):

The study Meyer et al performed study to understand the proteolytic cleavage of viral and cellular

proteins in two cell line models (Vero6 and A547-ACE2) of SARS-CoV-2 replication using mass spectrometry.

Major Comments:
1. Though the studies were performed in a logical way the major criticism is the choice of the cell lines. It is known that viral adaptation and proteolytic cleaves are cell type dependent. A recent study showed that "Blocking serine proteases, but not cathepsins, effectively inhibited SARS-CoV-2 entry and replication in human airway organoids" study by Mykytyn et. al.eLife (authors forgot to quote that). For proteolytic cleavage both the cell lines are not valid. I therefore request authors to have perform the experiments in organoids or cell model Calu-3 which will provide better physiological understanding.

2. The in vitro inhibition experiments are already reported by other researchers in other cell lines. However study has shown the the inhibition propensities were different in cell lines and the classic example was Hydroxychloroquine. A more mechanistic studies could be perform to understand the mechanism of the inhibition. Is it through the direct activity or through host directed therapies.

Minor Comments:
Authors have not quoted several papers in the field which showed similar results. It will be good to give credit those who have reported earlier.

Reviewer #3 (Remarks to the Author):

Major concern:
• Being an interesting and technically very sound study, it was performed on cellular models and in this respect, I think the overall viral load, when too high compared to that found in infected cells in patients might have tilted the balance such that quite some bystander substrates were here found cleaved as the viral protease activity was too high. Actually, figures 2B-D triggered my concern as more cleavage events are seen in the Vero E6 cells that have higher viral loads. Is there any possibility of quantifying viral proteolytic activity/measuring the viral load in the cellular models and in infected cells in patients? This might be quite impossible, yet one must keep this in mind when translating data from cellular infection models to infected patients and, in this respect, the data appear to be a bit oversold and, for sure, the manuscript title must be toned down.
Minor concerns:
• For a protein processing event to lead to/alter a cellular signalling event, one could argue that a sufficient fraction of protein processing is needed. Have the authors attempted to quantify the degree of protein processing? For instance, via Western blot in the infected cell systems? Such data could also be used to validate some of the described processing events.
• Lines 80-87: whilst I do agree with the authors' reasoning, maybe it is worthwhile in this respect to consider different types of vaccines (mRNA-based, proteins, inactivated virions)?
• Figures S1A-D: it is quite hard to distinguish the zero and six hour time-points of infection (colours are too alike).
• Lines 170-173: especially in cell cultures, 50% of the identified blocked N-termini being blocked with a TMTpro tag seems like a pretty high number. Any explanations for this? Could this be due to the fact that upon cell lysis, proteases were not efficiently inhibited giving rise to a lot of "background" cleavage events or is this the effect of the TMTpro tag that somewhat boosts peptide signals?
• Figures 1E-F: quite some neo-N-termini seem to be enriched in the mock sample, which appears counter-intuitive. Have the authors mined these N-termini and could they provide an explanation for this observation, as these are not further discussed, yet appear interesting.
• Line 211: change protoforms for proteoforms.
• Lines 272-289: can it be ruled out that the peptide starting at Q14 reflects an alternative translation initiation event rather than a cleavage event?
• General comment: for quite some of the viral proteins (especially the spike protein), quite some naturally occurring mutations have recently been described. Have the authors checked if some of these mutated sites were found processed in their study or a close to such processed sites?

<u>Response to reviewer comments:</u>

We thank the reviewers for their constructive recommendations, resulting in the attached significantly revised manuscript. In line with the reviewer suggestions, we have improved the data presentation, provided further computational/pathway analysis of our n-terminomic data and substrate cleavage, and performed additional experiments to further support the conclusions of the pseudovirus and siRNA experiments. Below, we have provided the reviewers comments in blue, and our point-by-point responses in black.

Yours sincerely
Dr Ed Emmott

Reviewer #1 (Remarks to the Author):

Meyer et al. studied the proteolytic cleavage of cellular and viral proteins in vivo upon SARS-CoV-2 infection of A549-ACE2 and Vero E6 cells by using an N-terminomic mass-spectrometry approach. Key findings were validated and inhibitors which target the proteolytically cleaved cellular proteins SRC and MYLK were active against SARS-CoV-2. Overall, the study represents a rich resource, which adds significant knowledge that can be further explored to identify novel regulatory mechanisms of virus infections and SARS-CoV-2 in particular. A limitation of the study are functional analyses regarding the individual cleavage products identified. The authors use mutational analysis close to the cleavage sites to study S-specific fragments but the resulting data is difficult to interpret. Moreover, I have some concerns regarding the functional analysis after siRNA treatment which would require additional supporting data. I also feel that the use of pleiotropic kinase inhibtors does not help to link the occurrence of cellular protein fragments to pro/antiviral functionality. Still, given the MS dataset and the novelty of the data provided I feel that this manuscript is interesting and should be revised.
We thank the reviewer for their comments on the rich resource this dataset provides, and novelty of the data with respect to both virus infection in general and SARS-CoV-2 in particular. We address their specific points of concern below.

Major comments:
1. While the authors provide a detailed analysis of cellular proteins that are potentially cleaved by viral proteases, they barley analyze the global pattern of neo-N-termini of cellular proteins that change upon virus infection. For example, activation of signaling cascades involved in innate immune responses or various stress-activated pathways might also lead to an increased cleavage of specific cellular proteins and hence produces neo-N-termini upon virus infection.

a. The authors should provide a more global analysis of their mass-spectrometry data including, for example pathway enrichment analysis, to potentially identify protease substrates that belong to the same pathway and could thereby indicate viral perturbations on pathway level.
We thank the reviewer for the suggestions and have expanded our analysis to incorporate GO term enrichment, motif analysis, as well as associating neo-N-termini with causal proteases to investigate protease activation/perturbation. Motif analysis is now included within the Figure on cellular substrates (Fig. 4), and the analysis of causal proteases and GO enrichment is included as supplementary figures S10-12. GO analysis did not identify activation of signaling cascades/stress-activated pathways, but did identify some shared features in both cell lines, notably mitochondria and cytoskeletal changes.

b. Additionally, do the authors detect an overrepresentation of amino acid sequence motives on neo-N-termini that would suggest an activation of specific cellular proteases upon virus infection?
We have moved the figures showing overrepresentation of Mpro consensus cleavage sites compatible with neo-N-termini to the supplementary data, and have included motif analysis as recommended by the reviewer. We see overrepresentation of serine at the P' side of the cleavage sites. As mentioned in response to point a), a subset of the cleavage events have annotated causal proteases, but systemic enrichment of substrates indicating regulation of

cellular proteases was not observed so we did not unambiguously identify the activation of specific cellular proteases.

2. Figure 1E, F present one of the most interesting datasets but the data is in my opinion not well presented.
a. mention q-value and fold change cutoffs (dashed lines) in legend

We have clarified q-value and the fold-change cutoffs in the legend.

b. What are purple dots? Please clarify
This was a display artifact derived from the viral markers also being hits, so overlaying the red and blue markers created the purple. This has been corrected and viral markers now display correctly as blue markers.

c. please provide an enlarged version of these volcano plots where the most significantly changing neo-N-termini are labeled
As requested, Fig. 1 has been revised to enlarge these volcano plots, with the top significantly changing neo-N-termini labelled.

d. please provide a scatter plot comparing the log2 fold change of neo-N-termini between A549 and Vero cells and comment on shared and unique neo-N-termini. It would be re-assuring that similar peptides are identified in the individual datasets.

We have included the requested scatter plots in the supplementary data (Fig S2). We made an explicit design choice when performing these experiments to have TMT-based quantification to maximize reproducible quantification within each cell line, rather than between them and as such, common peptides identified between the datasets represent only a subset of the data due to the data-dependent approach used to operate the mass spectrometer.

Those showing the strongest correlations are viral proteins. We have expanded on this in the text, and also highlight that given these cell lines reflect both different species and tissues where both total protein abundance and PTM state may be under different regulation and so this would be expected to reduce correlations.

3. The inhibition of proteases followed by N-terminomics is interesting. However, the authors correctly mention in line 375 that lack of TMPRSS2 in standard Vero-cells and likely also in A549-Ace2 cells. Can the authors please provide functional data that TMPRSS2 is active in their cell types? Otherwise the interpretation is very difficult – i.e. it is not poissible to functionally link the inhibitor data to activity of the inhibited proteases and phenotypes observed in the further experiments.

In line with multiple publications in the literature (e.g. Hoffman et al. 2020, Cell), including from the Olivier Schwatz lab (Buchrieser et al. 2020 EMBO J.), from which we sourced these cell lines, we do not believe TMPRSS2 is present or active in significant amounts in the Vero E6 or A549-Ace2 cell lines, though there is some reported basal expression at least at mRNA level in A549 (Di Ma et al. 2020, Eye). Camostat was thus included as a control which we have clarified in the text. As such we believe where we observed impact of Camostat in our dataset, this is most likely attributable to inhibition of other proteases, such as trypsin on which it has previously be reported to act in the low nanomolar range (e.g. 50nM Senokouchi et al. 1995, J. Med. Chem), and upon which its current clinical use in treatment of pancreatitis is based.

We would also highlight the timing of inhibitor addition: unlike most studies, we deliberately added inhibitors 12h post-infection as we were not aiming to inhibit viral entry. Instead, we wished to inhibit proteolytic cleavage of viral proteins late during infection, aiming to identify cleavage sites which could be attributed to cathepsin/calpain cleavage.

We have clarified this in the text. Entry into both cell lines is published as being cathepsin-dependent, consistent with the cathepsin inhibitor data we obtained using calpeptin. For the pseudovirus experiments, which seek to investigate potential roles of cleavage sites in cell entry, we used 293-Ace2 cells either further supplemented or lacking TMPRSS2, thus controlling for TMPRSS2 activity.

4. The pseudotyping experiment is not well described in text and figure legends and is somehow difficult to interpret. There is a lot of room for interpretation of this result, which – to the credit of the authors – they provide. However, it would be easy to gain further insights and support of these data with moderate effort:
a. Can the authors please provide an exemplary FACS blot comparing the most prominent candidates (e.g. V635G vs wt) (e.g scatter blots or MFI histogram)? This would be very reassuring on the effects shown.

We now provide representative scatter plots including the gating strategy for all of the spike pseudoviruses under investigation in both 293-Ace2 and 293-Ace2-TMPRSS2 cells as supplementary figures S7, S8.

b. It would be interesting to see the expression levels of transfected S mutants in 293T (producer) cells. This could indicate whether the introduced mutations would affect protein expression already in the producer cell or whether there is an issue on trafficking of the protiens. How do the different mutations localize intracellularly?
We performed a comparison of the relative levels of spike expressed on the surface of producer cells and after staining for intracellular spike. In line with our original data from Figure 3, only the 671 single/double mutants show reduced levels of S on the cell surface. In these samples, total levels of S observed following intracellular staining are similarly decreased with samples maintaining a similar surface:total ratio of signal, suggesting a defect in protein stability, which we mention in the text, rather than trafficking (Fig S9A).

c. While lentivector particles can be obtained in the absence of glycoproteins, interpretation of infectivity is depending on the presence of S on the lentivector pseudotypes. If the authors want to make statements on different infectivity (or loading of S on lentivectors) titrations are be required. The authors should use different doses of pseudotyped virus for the assay in order to allow proper appreciation of the effect.
We have followed the reviewer's suggestion and assessed the fraction of GFP+ cells for all mutants, following a 2-fold dilution series (0.25-2ug p24 equivalent) of the pseudotyped lentivectors (Fig S9B). The normalized infectivity we observed follows the same pattern as we originally observed in Fig. 3, at all inoculum amounts.

5. I am a bit concerned regarding the siRNA data even though this data is well controlled per se. Despite the surprisingly high knockdown efficiency (particularly considering transient knockdown in A549 cells, which are often not so easy to target) no toxicity of gene depletion is detected. However, some of the candidates have been shown to be essential for growth of eukaryotic cells (e.g. e.g. NUP107, PAICS, PNN and others/ doi:10.1007/s11010-010-0494-6, doi: 10.1186/s40659-018-0172-9, doi: 10.1242/jcs.100859). It is further surprising that all (!) tested candidates have a similar (inhibitory) effect. Even though the results are clear and appear controlled I feel that some additional proof that the effect is not simply due to reduced cell viability would be required. I would, for instance be more convinced if another readout that assures the viability of cells at the end of the experiments (e.g. by imaging and metabolic assays) would be used. It would, for instance be expected
that the highly reduced virus load also reduces cell cytotoxicity imposed by the virus in knockdown cells as compared to control conditions.

We thank the reviewer for this suggestion, we have addressed their concerns in two ways: we have performed the imaging-based viability analysis (counting DAPI-stained nuclei at the final timepoint) they suggested for all knockdowns in uninfected, and infected cells (Fig S18D,E). In line with ours, and the reviewer's expectations, the reduced virus load does permit higher cell viability compared to mock, for example in the case of GOLGA3, though this is not a significant increase for all targets. While siRNA KO efficiency is high at the time of infection (24h post-transfection), it is also not total and represents a transient knockdown, which likely explains why siRNA depletion of those proteins the reviewer mentions (NUP107, PAICS, PNN) are not lethal, and indeed show a small (albeit with the exception of PNN) though not significant increase in their viability compared to the control in the imaging-based assay in infected cells.

As an additional control, we also sought to add data on previously reported proteolytic targets of the SARS-CoV-2 proteases which did not show up in our analysis as their digestion with trypsin would not produce suitable length peptides for identification: IRF3 and TAB1 (Fig S18). For IRF3, siRNA knockdown was previously reported (Xin Yin et al. 2020, Cell Reports) as showing a small, but reproducible *increase* in viral titres, i.e. the opposite phenotype to all of our novel substrates.

In our hands, there was a visible albeit not significant increase in viral titres when calculated as PFU/mL on a log scale (i.e. absolute numbers) as we have done in a new supplementary figure for consistency with our other data throughout the manuscript. However, if we instead calculate our titres as relative values vs. the control, we do observe a significant (two-tailed unpaired t-test, $p < 0.01$) increase as shown in the figure below. The original Xin Yin et al. manuscript used a slightly different measure of titres: they calculated this as the fraction of cells staining +ve for nucleocapsid, and obtained values of ~55% for IRF3 siRNA-treated vs. ~20% for control-treated cells (Yin et al. Cell Reports, Fig. 5C. Their model was also different (Calu-3), but the overall difference: a approximate 3-fold increase remains the same and consistent with our observation. We believe these further data convincingly show that the inhibitory impact of siRNA depletion we observe for our novel substrates is not an artifact of our system. All these data, with the exception of the figure below is included as an additional supplementary figure (S18).

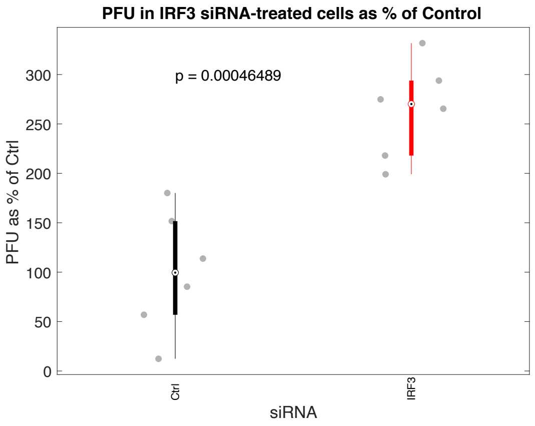

For review: siRNA depletion of IRF3 significantly increases SARS-CoV-2 titres by 72h post-infection as a % of titres from control-treated cells ($p<0.05$, two sample t-test).

Minor comments:
• General comments
o Add number and type of replicates for each experiment in figure legend
Figure legends have been updated with number and type (in all cases biological) of replicates.

o Always mention exact type of statistical test (e.g. two-sided unpaired student's t-test) in text/figure legends
We have gone through the text, and figure/supplementary figure legends to correct this omission.

• Even though the authors mostly focus on cleavage products it would be good to also comment whether non-canonical translation start sites could contribute to the observed protein patterns.
In line with R3's similar comment, we have expanded on this in the text. The .fasta database we used included all the proteins from Finkel et al. (2020) who applied ribo-seq to explicitly identify non-cannonical translation events. We did not unambiguously identify any of these non-cannonical translation events. Potentially due to low abundance, or simply not sampling the requisite peptides to distinguish extended or truncated versions or the relevant SARS-CoV-2 proteins. While we cannot exclude the possibility that some of our neo-N-termini represent non-cannonical translation events, we believe this this is a less likely explanation than proteolytic cleavage, given their absence from the Finkel et al dataset.

• Line 72
o Please use the appropriate gene and (protein) name from uniprot, e.g. Furin (FURIN) and Transmembrane protease serine 2 (TMPRSS2)
We thank the reviewer, and have corrected all references to FURIN and TMPRSS2 in the text.

• Line 192
o Please specify the exact type of t-test and multiple hypothesis test used
We thank the reviewer for spotting this, and in line with Nature Comms editorial policy have amended this throughout.

• Figure 1 and 6
o Please unify y-axis label by using PFU/ml in Figure 1 and introduce PFU in the legend
We thank the reviewer, and have amended Figure 1 to PFU/mL and defined PFU in both figure legends.

• Figure 1 legend
o Please introduce abbreviations in legend, e.g. bRP Fractionation in Figure 1
We have amended the figure legend to expand this abbreviation.

• Figure 2B-D
o Please label all neo-N-termini that are mentioned in the text
We have labelled those most prominently discussed in the text: within Fig 2D – these being 14, 260 and 637 which are prominently discussed later in the pseudovirus section.

To ensure continued readability of the figure, given a majority of the sites in Fig 2B/C are mentioned briefly in the text, we limited the labeling of datapoints to significant changes that are discussed in more depth. This is especially true for e.g. N where they neo-N-termini group in clusters. All viral neo-N-termini along with their intensity as well as further information is included in the supplementary tables on identified viral neo-N-termini so this data exists in the manuscript in a more readable and usable (.csv) format for reuse and reader exploration.

• Figure 2G
o May rather be provided as supplement figure since it is too technical for figure 2
As suggested, the MSMS spectrum has been moved to a supplemental figure (Fig. S4), alongside a MSMS spectrum for the spike signal peptide pyroglutamate-modified neo-N-terminus.

• Figure 3B
o Right y-axis label formatting error
Additional spaces in the Right Y-axis label have been removed.

• Figure 5B scale
o
Scale has been adjusted for figure 5B/C so that ticks encompass the full data range. Also the label for Fig 5C has been amended to PFU for consistency with Figures 1/6.

•Line 800-801
o Remove "…addition of TMPRSS2 was induced by…"
We thank the reviewer for spotting this, and have amended this sentence.

• Line 843
o (REF) missing
Thank you, the missing reference (Hughes et al. 2019, Nature Protocols) has now been included.

• Line 909
o Unit missing for NCE, 30%?
Thank you, this has been corrected to 30%.

Reviewer #2 (Remarks to the Author):

The study Meyer et al performed study to understand the proteolytic cleavage of viral and cellular proteins in two cell line models (Vero6 and A547-ACE2) of SARS-CoV-2 replication using mass spectrometry.

Major Comments:
1. Though the studies were performed in a logical way the major criticism is the choice of the cell lines. It is known that viral adaptation and proteolytic cleaves are cell type dependent. A recent study showed that "Blocking serine proteases, but not cathepsins, effectively inhibited SARS-CoV-2 entry and replication in human airway organoids" study by Mykytyn et. al.eLife (authors forgot to quote that). For proteolytic cleavage both the cell lines are not valid. I therefore request authors to have perform the experiments in organoids or cell model Calu-3 which will provide better physiological understanding.

We thank the reviewer for the comments regarding how the study was logically performed, and for highlighting the Mykytyn et al. study which we have been happy to include in this revised manuscript. We have endeavored to keep this study updated with the latest literature since its initial preprinting in September 2020, and this study published in January 2021 was unfortunately missed. We apologise to any other colleagues whose work has been inadvertently omitted.

The study referenced and others in the literature mention proteolytic cleavage of spike, an important process during virus entry. However, as our study highlights this is far from the only proteolytic process occurring during SARS-CoV-2 infection. There is no current reason to believe that proteolysis of cellular proteins by viral proteases, or that indeed cleavage of viral proteins other than spike should be impacted by lack of TMPRSS2 in the lines we have chosen. The few sites we highlight in spike (e.g. the signal peptide cleavage site and 637) have either been also identified in other systems (albeit overexpression) or we have shown are cathepsin-dependent and analysed in more detail in 293-Ace2 and 293-Ace2-TMPRSS2 in pseudovirus assays, thus controlling for the influence of TMPRSS2.

We agree with the reviewer that cleavage phenotypes can vary between cell lines, and for this reason we have gone further than most published SARS-CoV-2 proteomics studies in our use of two of the most widely-used cell lines for SARS-CoV-2 studies for our characterization. While no cell line or organoid model will ever perfectly recapitulate *in vivo* biology, we would disagree that the cell lines we have selected are 'not valid', or that by contrast organoids or Calu-3 represent a sole 'valid' model. This is especially true for a virus capable of infection and causing pathology in a range of organs far from the lung *in vivo*: features that would be missed with an exclusive focus on Calu-3 and lung organoid models.

It is also vital that results should be comparable with those generated from other labs. VeroE6 and Ace2-A549 cells although imperfect represent some of the most highly studied, and importantly: accessible models for SARS-CoV-2 (e.g. Gordon et al. 2020, Nature; Bouhaddou et al. 2020, Cell; Davidson et al. 2020, Genome Medicine), and indeed other corona- (e.g. avian coronavirus: Emmott et al. 2010, Mol. Cell. Proteomics), and respiratory viruses (e.g. RSV: Munday et al. 2010, Mol. Cell. Proteomics). Even more tellingly, in spite of their source from different species and tissues, we identified shared viral and cellular features of infection between A549-Ace2 and Vero E6 cells. Their use in our study better enables other groups to make full use of the resource this paper represents.

2. The in vitro inhibition experiments are already reported by other researchers in other cell lines. However study has shown the the inhibition propensities were different in cell lines and the classic example was Hydroxychloroquine. A more mechanistic studies could be perform to understand the mechanism of the inhibition. Is it through the direct activity or through host directed therapies.

We agree with the reviewer that inhibitor results can be cell line-dependent, the example they raise was a result from Vero E6 cells and indeed one of they key reasons why we focused on the lung Ace2-A549 cell line for the inhibitor studies. We have highlighted where our inhibitor results overlap with those in other studies, and our limitations section makes clear we believe it is important that validation should be conducted in other cell line and in vivo models prior to any suggestion of use in patients.

We agree that a mechanistic followup of these inhibition targets is an exciting area for future studies. However this falls outside the scope of the work we present here, and strongly believe would complicate and hinder the readability of this already lengthy manuscript.

Minor Comments:
Authors have not quoted several papers in the field which showed similar results. It will be good to give credit those who have reported earlier.

As mentioned in response to point one, we have updated our manuscript with new work that has emerged since our study was first released. We apologize to any colleagues whose work we have missed.

Reviewer #3 (Remarks to the Author):

Major concern:
• Being an interesting and technically very sound study, it was performed on cellular models and in this respect, I think the overall viral load, when too high compared to that found in infected cells in patients might have tilted the balance such that quite some bystander substrates were here found cleaved as the viral protease activity was too high. Actually, figures 2B-D triggered my concern as more cleavage events are seen in the Vero E6 cells that have higher viral loads. Is there any possibility of quantifying viral proteolytic activity/measuring the viral load in the cellular models and in infected cells in patients? This might be quite impossible, yet one must keep this in mind when translating data from cellular infection models to infected patients and, in this respect, the data appear to be a bit oversold and, for sure, the manuscript title must be toned down.

We appreciate the reviewers comments on the interest and technical soundness of the study. We have understood their concerns as having three key aspects: 1) The multiplicity of infection in a cellular model and 2) the possibility of correlating cleavage in these models with those seen in infected patients. 3) Overselling/title. I will address these in turn.

1) Viral load in these cell culture models (and model suitability).

To confirm, we used a multiplicity of infection of 1 in our study; reflecting a 1:1 ratio of infectious virus to cells. This would be expected to result in ~63% of cells becoming infected with one or more infectious particles in this first round of infection, and we only see infectious virus being produced in the very final timepoint. This is not a high multiplicity of infection by most published standards in virology where MOIs of 1, 3 or 10 are common. While we do not directly relate our observations to those observed *in vivo*, after the initial infection event of a single- or few cells with a small infectious dose, *in vivo* multiple studies suggest that neighboring cells may receive extremely high infectious doses, one example being guinea pig models of influenza virus infection, suggesting *in vivo* MOIs are established comparable to in vitro MOI of ~5-15 (Brooke (J. Virol) 2017, Brooke et al. (2014) PNAS. This view is also supported by *in vivo* imaging studies of SARS-CoV-2-infected ferrets which show dense foci of infection (Zaeck et al. (2021) Viruses).

2) Potential for comparing cleavage in these models with that in patients. This is not something we have attempted, and we agree with the reviewer that we do not believe this to be readily possible. The manuscript includes a limitations section at the end of the discussion making clear that these data are a very first step on the way to benefitting infected patients and that much further work is required before any drugs or therapies making use of these findings can be applied clinically. We have extended this limitations section to be more specific about the specific cell lines used and importance of *in vivo* validation.

With regard to the levels of infection in the different systems and the observed cleavage: we do not believe it is possible to directly correlate viral load and cleavage. However, it is possible to make some broad conclusions. The reviewer makes the point that a very high viral load could result in artifactual cleavage being observed through overexpression. In their study: Koudelka et al. 2020 Proteomics, the authors incubated lysed cell cultures with recombinant Mpro. They used this system to characterise the cleavage site specificity of Mpro, rather than for identifying cellular substrates per se. This system can also be considered as analogous to one where overexpression results in mis-compartmentalisation and the loss of the natural stoichiometry between Mpro and its substrates. We identified comparatively few substrates compared to this study, and saw low overlap between our substrates and theirs, with only GOLGA3 in common. If we had overexpression resulting in mislocalised or unexpectedly high levels of Mpro/PLP, we would expect to have seen greater overlap between these results.

With regards the title: In line with the reviewers suggestion we have revised the manuscript title to: Characterisation of protease activity during SARS-CoV-2 infection identifies novel viral cleavage sites and cellular targets with therapeutic potential.

We believe our data show convincing cellular targets which merit further investigation as targets for host-targeting antivirals, and we have shown a subset of inhibitors which do fulfil this role. We do make clear in the abstract and in the specific limitations section that these data should not be used to inform patient treatment without extensive further research including *in vivo* studies and as mentioned have extended our limitations section.

Minor concerns:
• For a protein processing event to lead to/alter a cellular signalling event, one could argue that a sufficient fraction of protein processing is needed. Have the authors attempted to quantify the degree of protein processing? For instance, via Western blot in the infected cell systems? Such data could also be used to validate some of the described processing events.

The fraction of protein cleaved has potential to influence downstream effects of protein cleavage, though this will depend on the specific products, and may only require a very minor fraction in the case of dominant cleavage products or specific subcellular niches being targeted, as we and other have published previously for proteolytic cleavage (e.g. Emmott et al. 2017, Mol. Cell. Proteomics), or occupancy of other PTMs.

To attempt to quantify the degree of protein processing, we have applied the HIquant approach for quantifying homologous proteoforms and modification site occupancy from quantitative proteomics data to our high confidence cellular cleavage events (Malioutov et al. 2019, Mol. Cell Proteomics doi: 10.1074/mcp.TIR118.000947). Use of this specific approach has permitted us to investigate the fraction of cleavage at each observed site where TMTpro labelling enabled relative quantification. This approach enabled us to investigate six of the cellular substrates cleaved by Mpro or PLP during viral infection, finding that the majority of the substrate is indeed cleaved. This is now included

as a supplementary figure (Fig. S14). As for the other substrates, clustering patterns in the unenriched (non-N-terminal) peptides indicate to us that potential PTMs/isoforms prevent accurate inference.

The HIquant (and similar approaches) require accurate relative quantification, and perform best when proteoforms (e.g. cleaved and uncleaved occupy a 1-100-fold range (Malioutov et al. 2019, Mol. Cell Proteomics). TMT and TMTpro reagents are known to suffer from ratio compression at very high fold-change, such as the dynamic ranges occurring for viral proteins throughout this timecourse. For this reason, use of HIquant to analyse the cleavage stoichiometry of viral proteins was not attempted as the inaccuracies this large dynamic range would introduce into the quantification would compromise the ability to accurately infer cleavage stoichiometry.

• Lines 80-87: whilst I do agree with the authors' reasoning, maybe it is worthwhile in this respect to consider different types of vaccines (mRNA-based, proteins, inactivated virions)?

We agree with the reviewer that the extent to which this will impact a vaccine will be dependent on the type of vaccine and have included an additional reference covering vaccine platforms and a further sentence expanding on this point.

• Figures S1A-D: it is quite hard to distinguish the zero and six hour time-points of infection (colours are too alike).

We agree with the reviewer and have changed the 6 hour timepoint in Figure S1A-D to black.

• Lines 170-173: especially in cell cultures, 50% of the identified blocked N-termini being blocked with a TMTpro tag seems like a pretty high number. Any explanations for this? Could this be due to the fact that upon cell lysis, proteases were not efficiently inhibited giving rise to a lot of "background" cleavage events or is this the effect of the TMTpro tag that somewhat boosts peptide signals?

We believe our results are in line with previous proteomics studies. The N-terminomics approach identifies all cleavage events, including for example removal of N-terminal methionine and signal peptide removal which affect a large fraction of the proteome. Unless the N-termini from such processes are modified e.g. by N-terminal acetylation or cyclization to pyroglutamate they are available for TMT labelling. The samples were lysed in SDS in the presence of high-dose protease inhibitors and immediately heated to 90C to minimize background proteolytic activity. This high % of labelled neo-N-termini is consistent with previous (non-TMT-based) versions of the HUNTER approach, which used dimethyl labelling (Weng et al. 2019, Mol. Cell Proteomics). Previous studies *in vivo* have identified over 47-70% of the proteome exhibiting proteolytic cleavage (auf dem Keller et al. 2013, Science Signaling, Lange et al. 2014 J. Proteome Res, Prudova et al. 2014 Blood)), and the proteins which we observe in our enriched samples are only a subset of the total proteome observed in the unenriched samples.

• Figures 1E-F: quite some neo-N-termini seem to be enriched in the mock sample, which appears counter-intuitive. Have the authors mined these N-termini and could they provide an explanation for this observation, as these are not further discussed, yet appear interesting.

We have mined neo-N-termini enriched and decreased for abundance for GO term/motif enrichment as part of our response to Reviewer #1 major comment 1. We do identify causal proteases for a subset, but do not observe systemic enrichment or diminished activity for specific proteases.

• Line 211: change protoforms for proteoforms.

Text corrected to 'proteoforms'.

• Lines 272-289: can it be ruled out that the peptide starting at Q14 reflects an alternative translation initiation event rather than a cleavage event?

Generation through an alternative translation initiation event cannot be ruled out, but we consider it unlikely especially given this site has not featured in published riboseq data (e.g. Finkel et al. 2020 Nature) which explicitly sought to identify alternate translation initiation events. We also note that further publications identifying this pyroglutamage modification at Q14 have now been released, albeit with spike overexpression in HEK293 or CHO cells, rather than infection and these references have been added to the revised manuscript. Considering the proximal nature of the predicted signal peptide cleavage site we concluded that this peptide starting at Q14 is most likely explained by signal peptide cleavage.

Our search .fasta file was constructed to include alternate translation initiation products/overlapping ORFs found in Finkel et al. We did not identify such alternate translation initiation products or overlapping ORFs in our work, likely due to their comparatively low expression levels, or where trypsin did not provide appropriate peptides to unambiguously identify such proteins.

• General comment: for quite some of the viral proteins (especially the spike protein), quite some naturally occurring mutations have recently been described. Have the authors checked if some of these mutated sites were found processed in their study or a close to such processed sites?

We thank the reviewer for this suggestion and have now included such analysis. We used covariants.org (accessed 24th May 2021) as a source for characteristic mutations found in variants of interest or concern. These annotated results identifying the closest neo-N-termini to each mutation have been added as a supplementary table. Out of the 107 unique mutations from the 8 variants of concern/under investigation, there are 9 mutations within 5 amino acids of our identified viral neo-N-termini, and 17 within 10 amino acids. This has been discussed further within the results and discussion sections, and in a new supplementary figure (S6) and two supplementary tables (S6, S7).

Notes on proteomics data access:

All proteomics data are deposited in the PRIDE repository. We had previously for reviewer convenience included the reviewer usernames and passwords in a supplementary table within the manuscript– these will be redundant upon publication as EBI PRIDE will automatically release the data for general access upon publication. For peer review, this table is included here – but is no longer included in the manuscript. The accession numbers are included in the ‘Data availability section’ as required.

Dataset:	PRIDE accession:	Reviewer Username:	Reviewer Password:
A549-Ace2 Unenriched	PXD021145	reviewer34884@ebi.ac.uk	ZKTFOUZA
A549-Ace2 Enriched	PXD021152	reviewer62064@ebi.ac.uk	xBgebTgO
A549-Ace2 Enriched (Variable Search)	PXD021402	reviewer_pxd021402@ebi.ac.uk	iSbBtAdt
Vero E6 Unenriched	PXD021154	reviewer98284@ebi.ac.uk	x3fU4szp
Vero E6 Enriched	PXD021153	reviewer53878@ebi.ac.uk	BiDiQ0VG
Vero Enriched (Variable Search)	PXD021403	reviewer_pxd021403@ebi.ac.uk	x7ZdiAt6
Vero Inhibitors Unenriched	PXD023539	reviewer_pxd023539@ebi.ac.uk	qcWplUZ5
Vero Inhibitors Enriched	PXD023538	reviewer_pxd023538@ebi.ac.uk	17XQ2usb
Vero Inhibitors Enriched (Variable Search)	PXD023540	reviewer_pxd023540@ebi.ac.uk	ktmN3bbU

Reviewer comments, second round –

Reviewer #1 (Remarks to the Author):

The authors have adequately addressed the comments I raised in my first review.

Reviewer #2 (Remarks to the Author):

I would like to thank the authors for taking time to address my previous comments. However still I believe that choice of cell lines is not appropriate. Though author argued using the references Gordon et al. 2020, Nature; Bouhaddou et al. 2020, Cell; Davidson et al. 2020, Genome Medicine. Those studies were performed when we don't have much knowledge about the virus and we are learning everyday about this virus. Therefore appropriate experimental model is important for improving knowledge in the field.
I would like to quote from Hoffmann et al 2020, nature "that chloroquine and hydroxychloroquine will exert no antiviral activity in human lung tissue and will not be effective against COVID-19, in keeping with the results of recent clinical trials14,15. Moreover, our results highlight the fact that cell lines that mimic important aspects of respiratory epithelial cells should be used when analysing the antiviral activity of compounds that target host cell functions."
Hoffmann et al 2020 published after Gordon et al.

Response to reviewers:

Reviewer comments are reproduced below in blue, our responses in black.

Reviewer #1 (Remarks to the Author):

The authors have adequately addressed the comments I raised in my first review.

We thank the reviewer for their previous suggestions and comments and their response.

Reviewer #2 (Remarks to the Author):

I would like to thank the authors for taking time to address my previous comments. However still I believe that choice of cell lines is not appropriate. Though author argued using the references Gordon et al. 2020, Nature; Bouhaddou et al. 2020, Cell; Davidson et al. 2020, Genome Medicine. Those studies were performed when we don't have much knowledge about the virus and we are learning everyday about this virus. Therefore appropriate experimental model is important for improving knowledge in the field.
I would like to quote from Hoffmann et al 2020, nature "that chloroquine and hydroxychloroquine will exert no antiviral activity in human lung tissue and will not be effective against COVID-19, in keeping with the results of recent clinical trials14,15. Moreover, our results highlight the fact that cell lines that mimic important aspects of respiratory epithelial cells should be used when analysing the antiviral activity of compounds that target host cell functions."
Hoffmann et al 2020 published after Gordon et al.

Fundamentally, we agree with the reviewer that selection of model system is an important choice, and can influence experimental results. For the reasons we stated in our previous response, we believe the systems we have chosen are appropriate and suitably controlled for TMPRSS2 expression in the specific cases where this was relevant.

Regarding antivirals, as we stated, we performed all our siRNA/antiviral assays in A549-Ace2 cells, a human alveolar basal epithelial cell line expressing the Ace2 receptor, which given the above clearly meets the criteria they have quoted from Hoffman et al 2020: ‘cell lines that mimic important aspects of respiratory epithelial cells should be used’. We do not believe results in any single-cell line or model system should be taken as gospel, and our discussion does mention (in agreement with the reviewer), that validation in further cell lines/model systems is an important next step prior to any suggestion of use *in vivo* or clinical trials.

We are adding a further line to the discussion on the appropriateness of the cell lines we have chosen, however for the reasons we have stated in our prior response, believe our selection is suitable for the experimental questions we have posed. We fully support Nature Communications policy of transparent peer review where (with reviewer consent) the journal provides the

anonymised peer review process for readers, supporting them in using their own judgement as to our manuscripts particular strengths and weaknesses.
